# The sucrose non-fermenting 1-related kinase 2 gene *SAPK9* improves drought tolerance and grain yield in rice by modulating cellular osmotic potential, stomatal closure and stress-responsive gene expression

**DOI:** 10.1186/s12870-016-0845-x

**Published:** 2016-07-13

**Authors:** Avishek Dey, Milan Kumar Samanta, Srimonta Gayen, Mrinal K. Maiti

**Affiliations:** Advanced Laboratory for Plant Genetic Engineering, Advanced Technology Development Centre, Indian Institute of Technology Kharagpur, Kharagpur, 721302 India; Present address: Department of Human Genetics, University of Michigan, Ann Arbor, MI 48109 USA; Department of Biotechnology, Indian Institute of Technology Kharagpur, Kharagpur, 721302 India

**Keywords:** Abscisic acid (ABA), Drought tolerance, Gene silencing, Grain yield, Overexpression, Osmotic potential, Rice crop, SAPK9, Stomatal closure, Stress-responsive gene, Sucrose non-fermenting 1-related kinase 2 (SnRK2)

## Abstract

**Background:**

Family members of sucrose non-fermenting 1-related kinase 2 (SnRK2), being plant-specific serine/threonine protein kinases, constitute the central core of abscisic acid (ABA)-dependent and ABA-independent signaling pathways, and are key regulators of abiotic stress adaptation in plants. We report here the functional characterization of *SAPK9* gene, one of the 10 SnRK2s of rice, through developing *gain-of-function* and *loss-of-function* phenotypes by transgenesis.

**Results:**

The gene expression profiling revealed that the abundance of single gene-derived *SAPK9* transcript was significantly higher in drought-tolerant rice genotypes than the drought-sensitive ones, and its expression was comparatively greater in reproductive stage than the vegetative stage. The highest expression of *SAPK9* gene in drought-tolerant *Oryza rufipogon* prompted us to clone and characterise the CDS of this allele in details. The *SAPK9* transcript expression was found to be highest in leaf and upregulated during drought stress and ABA treatment. *In silico* homology modelling of SAPK9 with *Arabidopsis* OST1 protein showed the bilobal kinase fold structure of SAPK9, which upon bacterial expression was able to phosphorylate itself, histone III and OsbZIP23 as substrates in vitro. Transgenic overexpression (OE) of *SAPK9* CDS from *O. rufipogon* in a drought-sensitive *indica* rice genotype exhibited significantly improved drought tolerance in comparison to transgenic silencing (RNAi) lines and non-transgenic (NT) plants. In contrast to RNAi and NT plants, the enhanced drought tolerance of OE lines was concurrently supported by the upgraded physiological indices with respect to water retention capacity, soluble sugar and proline content, stomatal closure, membrane stability, and cellular detoxification. Upregulated transcript expressions of six ABA-dependent stress-responsive genes and increased sensitivity to exogenous ABA of OE lines indicate that the SAPK9 is a positive regulator of ABA-mediated stress signaling pathways in rice. The yield-related traits of OE lines were augmented significantly, which resulted from the highest percentage of fertile pollens in OE lines when compared with RNAi and NT plants.

**Conclusion:**

The present study establishes the functional role of SAPK9 as transactivating kinase and potential transcriptional activator in drought stress adaptation of rice plant. The *SAPK9* gene has potential usefulness in transgenic breeding for improving drought tolerance and grain yield in crop plants.

**Electronic supplementary material:**

The online version of this article (doi:10.1186/s12870-016-0845-x) contains supplementary material, which is available to authorized users.

## Background

Plants, being sessile are incessantly confronted by different environmental stresses, which include drought, high salinity, and extreme temperature, affecting both biomass productivity and grain yield of crops. To cope with such adverse multiple stresses, diverse molecular and physiological mechanisms have been evolved by the plant kingdom in general and a plant species in particular. Therefore, it is indispensable to comprehend the specific mechanism of responses by any crop species to such stresses, with the ultimate aim of improving crop performance. Rice (*Oryza sativa* L.), being one of the most important cereal crops, feeds more than half of the world population and is adversely affected by drought at the morphological, physiological and molecular level. The phytohormone abscisic acid (ABA), which is produced under the drought stress functions to regulate several developmental and physiological processes including seed maturation, germination, seedling growth and transpiration. The ABA level increases under water deficit condition in plants triggering stomatal closure and responses to stress tolerance [1]. Stress signals are recognized by specialized signaling pathways which transmit them to different cellular compartments, and the numerous evidences demonstrated that protein kinases play vital roles in the responses to such environmental stimuli [2]. The sucrose non-fermenting 1-related kinase 2 (SnRK2) family members, which function in diverse developmental processes in plants [3, 4], have been shown to be the positive regulators of plant response to abiotic stresses [5–8]. The members of SnRK2 family work at the merging point of the ABA-dependent and ABA-independent stress signaling pathways.

In *Arabidopsis*, ten members of the SnRK2 family have been identified and divided into three subclasses based on amino acid sequence similarity [9]. The subclass I comprises of kinases that are not activated by ABA, subclass II are either not activated or very weakly activated by ABA (depending on plant species). However, the subclass III is strongly activated by ABA. The amino acid sequences of all the SnRK2s can be divided into two regions, the highly conserved N-terminal kinase domain, and the C-terminal regulatory domain. The C-terminal domain contains stretches of acidic amino acids, either glutamic acid (E) (subclass I) or aspartic acid (D) (subclass II and III). Further, the C-terminal regulatory domain consists of two subdomains, subdomain I and subdomain II. The subdomain I which is required for activation by osmotic stress, independent of ABA, is present in all SnRK2 family members; whereas subdomain II is necessary for the ABA response and is specific to the ABA-dependent SnRK2s only. All the SnRK2 members, except SnRK2.9, were found to be rapidly induced by different osmolyte treatments, such as mannitol, sorbitol, sucrose or NaCl and few of them also by ABA [10]. The *Arabidopsis* OST1/SnRK2.6/SRK2E protein kinase has been shown to regulate the ABA-mediated stomatal closure and act upstream of reactive oxygen species production [6]. However, the *srk2e* (*ost1*) mutant in *Arabidopsis* has been found to be defective in ABA-induced stomatal closure and it showed a wilty phenotype [7]. The *srk2d*/*e*/*i* triple mutant (for SnRK2.2/SRK2D, SnRK2.6/SRK2E and SnRK2.3/SRK2I genes) of *Arabidopsis* displayed dramatically decreased drought tolerance and extreme insensitivity to ABA, as documented by defects in seed germination and seedling growth as well as decreased expression of ABA- and stress-inducible genes [11]. The knockout of these three genes, which belong to the subclass III of SnRK2 family, therefore, almost completely blocks ABA responses, demonstrating that they are the essential components of ABA-stress signaling pathway in *Arabidopsis* [3].

Eleven SnRK2 members designated as ZmSnRK2s have been identified in maize, and most of them are inducible by one or more abiotic stresses [12]. The ZmSnRK2.8 protein, which is highly homologous to *Arabidopsis* OST1, has been found to be involved in diverse stress signaling pathways, particularly in salt stress tolerance [12, 13]. In wheat, the first characterized SnRK2 member, *PKABA1* was reported to be induced by hyperosmotic stress, ABA, and multiple other environmental factors [14, 15]. Afterwards, three more wheat SnRK2 members viz., TaSnRK2.3, TaSnRK2.4 and TaSnRK2.8 have been characterized and found to be involved in development and abiotic stress tolerance [16–18]. Thus, substantial evidence showed that the SnRK2 protein kinases are involved in multiple environmental stress responses and all have potential biotechnological utility for generation of high yielding abiotic stress tolerant crops [16]. In vitro studies have documented that the ABA-activated SnRK2s phosphorylate the downstream target proteins in different plant species; and this phosphorylation is needed for the transcriptional activity of the individual target proteins, which in turn induce the expression of hierarchically downstream genes to mitigate the stress condition. This includes bZIP transcription factors, such as TRAB1 from rice [19], TaABF from wheat [20], and AREB1 from *Arabidopsis* [21], or RNA-binding VfAKIP1 proteins from *Vicia faba* [22].

In rice, the ten SnRK2 members, designated as SAPK1-10 (osmotic stress/ABA-activated protein kinase 1-10) have been identified. All of them were found to be activated by hyperosmotic stress, and SAPK8-10 were also induced by ABA [23]. The overexpression of *SAPK4* showed improved salt tolerance in rice [24]. In the domain swapping experiments using rice SAPKs, it has been observed that the grafting of the non-catalytic C-terminal region from SAPK8 (Glu-254 to Met-372), which contains the domain involved in ABA-responsive activation, onto the catalytic domain of SAPK2, which doesn’t contain the domain for ABA-responsive activation, is sufficient to confer ABA responsiveness in SAPK2 [23]. None of the three ABA-dependent and osmotic stress-activated SAPKs, i.e. SAPK8, SAPK9, and SAPK10, belonging to subclass III of SnRK2 family, has been functionally characterized by transgenic approach till date. Therefore, understanding the molecular basis of subclass III SAPK gene function in rice is necessary for the development of drought-tolerant transgenic rice or to design SAPK gene-based marker for molecular breeding of drought-tolerant rice cultivar. We were interested in the detailed characterization of the *SAPK9* gene and its functional role in drought tolerance through (i) expression profiling of the gene in selected drought-sensitive and drought-tolerant rice genotypes, (ii) determining the allelic polymorphism in the coding DNA sequence (CDS) of the gene in these genotypes, (iii) cloning the highly expressed *SAPK9* CDS from the drought-tolerant rice genotype *Oryza rufipogon* followed by assaying the kinase activity of the recombinant protein, and (iv) developing transgenic rice lines for overexpression and endogenous silencing of this particular gene. Transgenic experiments demonstrated that in contrast to silencing, the overexpression of SAPK9 increased drought tolerance and grain yield by adjusting osmotic potential and stomatal closure, thereby decreasing the cellular membrane damage and reactive oxygen species activity. In addition, the increased and decreased ABA sensitivity and ABA-responsive gene expression in transgenically overexpressed and silenced lines, respectively, confirmed that the SAPK9 is a positive regulator of ABA-dependent stress-responsive signaling pathway in rice.

## Methods

### Plant materials, growth condition and stress treatments

Nine cultivated *indica* rice (*Oryza sativa* L.) genotypes and two wild rice progenitors were selected for the experimental works. The cultivated rice genotypes comprised of drought-tolerant- Manipuri, Nagina22, Vandana and drought-sensitive- Swarna, HRC300, IR20, IR36, IR64, and IR72; while the two wild rice genotypes - *O. rufipogon* and *O. nivara* are drought-tolerant. The seed samples of all rice genotypes were provided by Indian Agricultural Research Institute, New Delhi. All the experimental works were performed using the seeds of same harvest and storage conditions, and rice genotypes were usually grown for 30 days inside the glasshouse, which was maintained at 25/28 °C temperature with 70 % relative humidity and 16/8 h light/dark photoperiod. Thereafter, the potted plants were transferred into the net-house. To analyse the effects of drought stress and ABA on the expression of *SAPK9* gene, rice seedlings were subjected to dehydration and ABA (100 μM) treatment for a period of 6–48 h and an untreated sample at 0 h was used as control. The transcript level of *SAPK9* gene was analysed from leaf tissues of rice genotypes grown under: (i) normal growth condition- designated as before stress (BS), (ii) withholding water supply for 8 days- referred as after stress (AS), and (iii) resuming water supply for 3 days after the drought stress period- referred as after recovery (AR). The experiments were performed at both vegetative and reproductive (or grain filling) stages. For T_1_ transgenic lines and non-transgenic (NT) plants drought stress treatment at the vegetative and early reproductive (panicle initiation) stages, and analysis of grain yield under drought condition were performed following the reported protocol [25].

### Cellular RNA isolation, first strand cDNA synthesis and real‑time PCR analysis

The total cellular RNA was isolated from rice leaves using RNeasy Mini Kit (Qiagen) following the vendor’s protocol. Total RNA (2 μg) was used to synthesize the first strand cDNA with the help of gene-specific 3'-primer and Transcriptor First Strand cDNA synthesis kit version 6.0 (Roche Diagnostics India Pvt. Ltd.) following the instructions of the manufacturer. For transcript expression profiling, real-time PCR was performed by means of SYBR green-based relative quantification method using RealMasterMix SYBR ROX (5 PRIME) kit in Eppendorf Realplex^2^ Master Cycler. The relative gene expression level was determined following the reported literature [26]. For each sample, three replicates were taken. In each case, rice polyubiquitin1 gene (*OsUbi1*) was used as internal reference. The different primers used for the real-time PCR are provided in Additional file 1: Table S1.

### Southern hybridization

Standard protocols were followed to isolate the genomic DNA from rice leaves and to perform Southern blot hybridization [27]. In brief, the *Hind*III-digested genomic DNA (12 μg) upon electrophoresis on agarose gel overnight, was transferred onto nylon membrane (Hybond-N^+^). A 470 bp DNA fragment from the middle of SAPK9 coding DNA sequence (CDS) from *O. rufipogon* was PCR-amplified using gene-specific primers SA9SF-SA9SR (Additional file 1: Table S1) and radiolabelled with P^32^-dCTP (3500 Ci/mmol) by random priming using rediprime II DNA labelling system (GE Healthcare, USA) following the vendor’s instructions. For autoradiography, the Cylone® Plus phosphor system (Perkin Elmer) was used to scan the multi-sensitive X-ray film (Perkin Elmer).

### Cloning and sequence analysis of the *SAPK9* CDS

The full-length CDS of *SAPK9* gene was PCR amplified using 1^st^ strand cDNA sample as a template from the drought-sensitive and drought-tolerant rice genotypes mentioned in plant materials section. The gene-specific primers SK9F and SK9R (Additional file 1: Table S1) were used for the PCR amplification in a thermocycler (Applied Biosystems) with the following thermal profile: an initial denaturation at 98 °C for 2 min, followed by 30 cycles of 98 °C-for 10 s, 62 °C for 15 s, 72 °C for 1 min and a final extension at 72 °C for 8 min. All the PCR amplicons (~1086 bp size) were individually cloned by blunt-end ligation into pUC18 plasmid digested with *Sma*I restriction enzyme. Positive clones were sequenced, and nucleotide polymorphism of all the CDSs were analysed. The multiple sequence alignment of the CDS-derived polypeptides obtained from all the genotypes was performed using the Jalview 2 software (http://www.jalview.org/Download).

### Phylogenetic analysis

Multiple amino acid sequence alignment of SnRK2 family proteins from *Arabidopsis*, rice, and a few other plant species were performed using the ClustalX2 (http://www.clusal.org/clustal2/). The phylogenetic tree was constructed using the neighbor-joining method in MEGA6 (http://www.megasoftware.net/mega.php). To evaluate the reliability of the tree, a bootstrap analysis was performed using 1000 replicates in MEGA6.

### Homology modelling

The homology model of SAPK9 was made using the MODELLER 9.15 (https://salilab.org/modeller/download_installation.html). The solved crystal structure of the OST1/SnRK2.6 protein from Arabidopsis (PDB ID: 3UC4) was taken as a template for constructing the *in silico* model of SAPK9. The resulting model was energy minimized using Insight II (2000.1, Accelrys Inc.) followed by stereo-chemical evaluation using MolProbity (http://molprobity.biochem.duke.edu/). The pictorial representations were prepared in PyMol (http://www.pymol.org).

### In vitro kinase assay

The recombinant pRSET plasmid (Invitrogen) containing the 1086 bp CDS of *SAPK9* in *Xho*I and *EcoR*I restriction sites allowed expression of 6xHis N-terminal tagged SAPK9 protein in *E. coli* BL21 (DE3) pLysE strain (Invitrogen) upon 1 mM IPTG induction. Similarly, first 900 bp (encoding 300-amino acid) from the *OsbZIP23* CDS of *O. rufipogon* [25] was cloned into a pRSET vector in *Bam*HI and *Eco*RI restriction sites, transformed and expressed in pLysE cells with 0.5 mM IPTG induction. The expressed proteins were purified in native condition and used for in vitro kinase assay. In vitro phosphorylation of generic substrate histone III (Sigma) was performed as described previously [28]. In vitro phosphorylation of OsbZIP23 was performed by incubating the individual reaction mixture for 5, 25 and 40 min at 25 °C following the above-mentioned protocol. The products were fractionated in 12 % SDS-PAGE and visualized by autoradiography.

### Preparation of constructs for overexpression and silencing of *SAPK9* gene, and development of transgenic rice plants

For the preparation of *SAPK9* overexpression (OE) construct, the full-length 1086 bp CDS of *SAPK9* was cloned (as *Sal*I-*Kpn*I fragment) from *O. rufipogon* under the *OsUbi1* promoter (as *Hind*III-*Sal*I fragment) and NOS transcription terminator (as *Kpn*I-*Eco*RI fragment) in the pCAMBIA1301 binary plasmid. Thus, the *SAPK9* OE genetic construct was prepared (Additional file 2: Figure S5a). On the other hand, the RNAi-mediated gene silencing (RNAi) construct of *SAPK9* was prepared (Additional file 2: Figure S5b) by cloning a 605 bp DNA fragment from 5′-part of *SAPK9* CDS in sense (as *Sal*I-*Bam*HI fragment) and antisense (as *Bam*HI-*Kpn*I fragment) direction flanked by an arbitrary linker DNA (having *Bam*HI site at both ends) under the *OsUbi1* promoter (as *Hind*III-*Sal*I fragment) and NOS transcription terminator (as *Kpn*I-*Eco*RI fragment) in pCAMBIA1301 binary plasmid. Drought-sensitive IR20 rice cultivar was genetically transformed separately with these OE and RNAi constructs by *Agrobacterium*-mediated transfer technique, as reported earlier [29]. The transgenic rice lines developed with the OE and RNAi constructs were designated as SAOE#1, 2, 3 etc. and RNAi#1, 2, 3 etc, respectively. The list of primers used for preparing genetic constructs is given in Additional file 1: Table S1.

### Western blot analysis of SAPK9 protein in transgenic lines and non-transgenic rice plants

Protein samples (40 μg each) isolated from the leaf tissues of transgenic lines and NT rice plants were used for western blotting experiment as per the method reported earlier [25]. The custom made 15-amino acid peptide (MERNAAGPLGMEMNC) from the N-terminal end of SAPK9 was used to raise the polyclonal antibody in rabbit (IMGENEX India). Affinity purified polyclonal antibody of SAPK9 was taken as primary antibody (with 1: 1000 dilutions). The monoclonal antibody of plant β-actin (Sigma, catalog no.-A0480) was used (with 1:500 dilutions) to detect actin protein in the samples as a loading control. Immuno-detection was performed using the Lumi-LightPLUS Western Blotting Kit (Roche Diagnostics India Pvt. Ltd.), as per the vendor’s protocol.

### Estimation of water loss rate, relative water content, proline content and soluble sugar content in leaf samples of transgenic and non-transgenic rice plants

Water loss rate (WLR) and relative water content (RWC) in the leaves of transgenic and NT rice plants were determined on the basis of the published protocols [30, 31]. From the leaves (50 mg) of transgenic lines and NT rice plants free proline content was estimated as per the published literature [32]. Similarly, total soluble sugar content in rice leaves (100 mg) was determined using anthrone reagent [33]. Consistent results were obtained from two sets of the experiment, and the result from one experimental set is documented here. Average of three replicates was calculated to represent each data point.

### Analysis of rice stomata by scanning electron microscopy

Scanning electron microscopy analysis of leaf samples from transgenic and NT rice plants (collected before and after drought stress) were performed following the described method [34]. The samples were mounted onto the sample stages and coating treatment was performed. The stomatal pictures were obtained using a scanning electron microscope (ZEISS, Germany).

### Estimation of malondialdehyde (MDA) content and detection of reactive oxygen species (ROS) in leaf samples of rice plants

Rice leaf (100 mg) of transgenic lines and NT plants was used to determine the MDA content following the reported method [35]. In vivo localization of ROS in intact leaves of the transgenic lines and NT plants were carried out following the method described earlier [36]. Consistent results were obtained from two sets of experiment and the result from one experimental set is documented here. Average of three replicates was calculated to represent each data point for MDA content.

### Subcellular localization of the SAPK9 protein

The SAPK9 CDS was cloned in frame at the 5′-end of the green fluorescence protein (*GFP*) gene at *Bgl*II and *Spe*I restriction sites under the control of constitutive CaMV35S promoter in the binary plasmid pCAMBIA1302. The list of primers used is given in Additional file 1: Table S1. *Agrobacterium-*mediated transformation of onion epidermal cells was carried out following the previously published protocol [37].

### Assay of ABA sensitivity during seed germination and post-germination stages

Sensitivity towards ABA during seed germination and post-germination (seedling) stages was analysed using the different concentrations of ABA (0, 1, 3, 6 μM) in the growth media following the reported protocol [25].

### Estimation of spikelet fertility and pollen maturation

The transgenic lines and NT plants were grown normally in PVC pipes. Upon attaining flowering stage, plants were subjected to drought stress for a period of 10 days followed by recovery until seed maturation occurred. The panicle weight and spikelet fertility were calculated in replicates. For pollen staining, 1 % I_2_-KI solution based method was followed as described previously [38].

### Accession numbers

Sequence information of the genes mentioned in this study is available in the GenBank database having their respective accession numbers: Rice polyubiquitin 1 (*OsUbi1*) gene (AF184279), *OsUbi1* promoter (AY785814), *SAPK9* CDS from wild rice *O. rufipogon* (KT387673), *OsRab16B* gene (AF333275), *OsRab21* gene (Y00842) *OsLEA3-1* gene (DQ789359), *OsbZIP23* gene (KP779638), *TRAB1* gene (BAD09357), *OsbZIP46* gene (ADK60888), *SAPK8* gene (AB125309), *SAPK10* gene (AB125311), *OsSLAC1* gene (LOC_Os04g48530.1) and *OsSLAC7* gene (LOC_Os01g28840).

## Results and discussion

### The single copy *SAPK9* gene displays differential transcript expression profile and allelic polymorphism in drought-tolerant and drought-sensitive rice genotypes

**Fig. 1:**
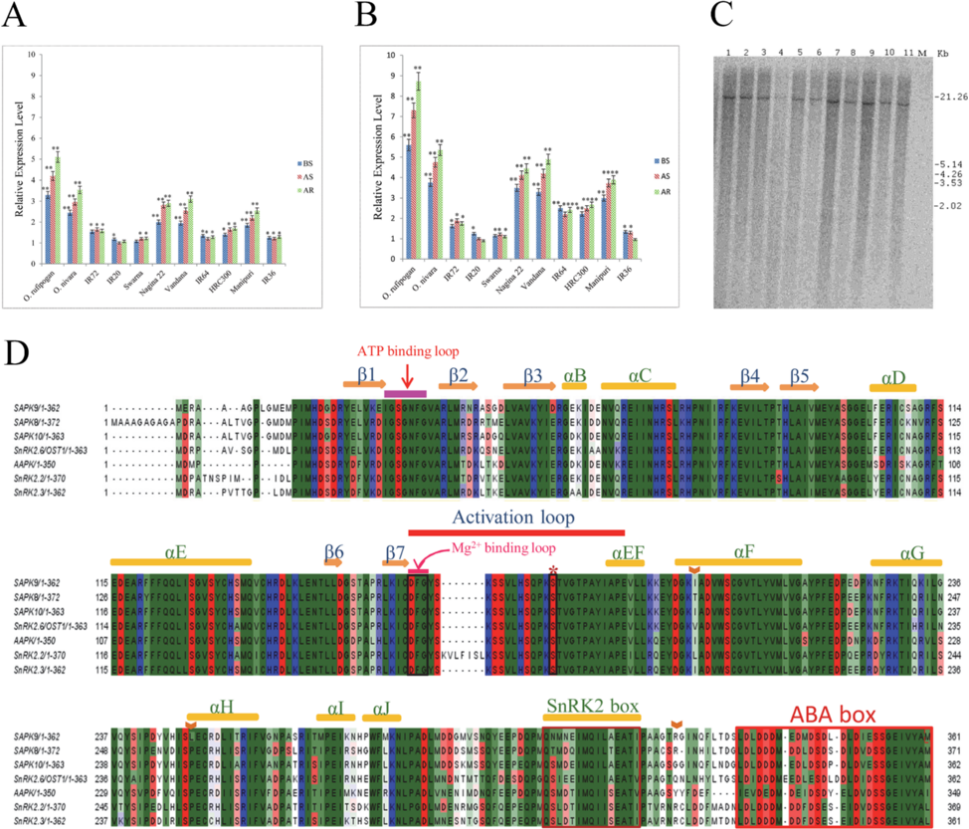
Expression profiling and copy number determination of the endogenous *SAPK9* gene in 11 selected rice genotypes. Real-time PCR analysis showing *SAPK9* gene expression in (**a**) vegetative stage and (**b**) reproductive stage (grain filling) in rice plants growing under the condition of before stress (BS), after stress (AS) and after recovery (AR). For internal reference, rice polyubiquitin1 (*OsUbi1*) gene was used. Error bars represent the mean ± SD of triplicate measurements. Student’s *t*-test was performed to find out statistically significant differences (* *P* < 0.05, **P < 0.01). **c** Southern hybridization blot depicting the presence of one copy of the endogenous *SAPK9* gene in all the rice genotypes examined. Lanes 1-11 represents *O. rufipogon, O. nivara,* Nagina22, Manipuri, Vandana, Swarna, IR20, IR36, IR64, IR72 and HRC300 genotypes, respectively. **d** Multiple amino acid sequence alignment of subclass III SnRK2 family members including SAPK9 protein for the prediction of secondary structures following the PROSITE ExPASy bioinformatics tool

The ability of a plant species to withstand drought stress condition is a genotype-dependent functional activity of the particular genes involved in the stress-adaptive pathways. Hypothesizing that the SAPK9 is a key player in drought tolerance, the relative expression level of *SAPK9* gene was analysed in nine selected drought-sensitive and drought-tolerant *indica* rice genotypes along with two wild rice progenitors during the vegetative and reproductive stages of growth through real-time PCR. For the analysis, first strand cDNA was synthesized from leaf total RNA of all the genotypes under the condition of before stress (BS), after stress (AS) and after recovery (AR). The real-time PCR analysis of BS, AS and AR samples revealed that the expression level of *SAPK9* transcript in *O. rufipogon* is substantially greater than the other drought-tolerant genotypes viz., *O. nivara*, Nagina22, Manipuri and Vandana in both vegetative stage (Fig. 1a) and reproductive stage (Fig. 1b). On the other hand, the *SAPK9* expressions in drought-sensitive genotypes viz., HRC300, IR20, IR36, IR64, IR72, and Swarna were comparatively much lower than the drought-tolerant genotypes in both the growth stages (Fig. 1a and b). It was also observed that the expression of *SAPK9* transcript is elevated in reproductive stage (Fig. 1b) compared to the vegetative stage (Fig. 1a). To check the influence of gene copy number if any, on the differential expression of *SAPK9* transcript in the rice genotypes under the same growth conditions, we performed Southern hybridization using *SAPK9* gene-specific probe. A single band observed in Southern blot indicated the presence of one copy of the endogenous *SAPK9* gene in all the rice genotypes investigated (Fig. 1c). The occurrence of a single copy endogenous *SAPK9* gene in the selected rice genotypes implies that the gene copy number does not influence the differential expression of *SAPK9* gene in drought-sensitive and drought-tolerant rice genotypes.

Single nucleotide polymorphism (SNP) in the CDS is of great importance in the crop improvement programme as the functions of several genes are known to be modulated by the associated SNPs leading to the differences in plant performance. Assessing genetic diversity of candidate gene sequences involved in stress-responsive pathways lead to the identification of specific alleles which are linked to particular agronomic traits of abiotic stress responses. Trait enhancing superior alleles, which are dominant and express better in wild species than cultivated varieties and are left behind during evolution and domestication of present-day commercial cultivars, can be transferred to elite genetic backgrounds for improvement of desirable traits of a particular crop species [39]. Therefore, it was of interest to investigate the presence of naturally occurring polymorphisms in the CDS of *SAPK9* gene in all the chosen rice genotypes.

For the analysis of natural allelic polymorphism, the full-length CDS of *SAPK9* gene was amplified through RT-PCR from total RNA of leaf tissues using two gene-specific primers. All the genotypes showed positive PCR amplification with an expected product size of ~1086 bp (Additional file 3: Figure S1). The sequence of each PCR amplicon was determined after cloning them individually. Three non-synonymous SNPs were detected among the *SAPK9* CDSs obtained from eleven rice genotypes upon multiple sequence alignment. The first non-synonymous SNP was found in the CDS of Swarna, creating the nucleotide modification (T to A) at the 593^rd^ position from the start codon, which led to the 198^th^ amino acid change from isoleucine (I) to asparagine (N) (Additional file 4: Figure S2). The resulted amino acid change was located in the αF-helix of SAPK9 protein (Fig. 1d). The 2^nd^ non-synonymous SNP was noticed in *O. rufipogon*, causing the nucleotide modification (C to T) at the 746^th^ position from the start codon with the 249^th^ amino acid change from proline (P) to leucine (L) (Additional file 4: Figure S2), and this was observed in the αH-helix of the SAPK9 protein (Fig. 1d). The 3^rd^ non-synonymous SNP, being observed in a few genotypes, resulted in the nucleotide modification (A to C) at the 970^th^ position from the start codon, which changed the 324^th^ amino acid from serine (S) to arginine (R). Serine is present at 324^th^ amino acid position in Swarna, IR20, IR36, IR64, HRC300, and Vandana; whereas arginine occurs at the same position in *O. rufipogon*, *O. nivara*, IR72, Nagina22 and Manipuri (Additional file 4: Figure S2). This last amino acid change was found to be located in between the SnRK2 box and ABA-dependent activation box (ABA-box) of SAPK9 protein (Fig. 1d). Various reports of non-synonymous SNPs leading to amino acid substitution in the CDS of drought responsive genes and their correlation with the particular trait have been published earlier [40]. However, the present study showed that the natural occurrence of three non-synonymous SNPs did not affect any important functional domains and also could not be correlated with the differential expression level of the *SAPK9* gene in the chosen drought-sensitive and drought-tolerant rice genotypes. Therefore, the highly expressed *SAPK9* CDS of wild progenitor *O. rufipogon* was selected for further functional analyses.

### The *SAPK9* gene from drought-tolerant *O. rufipogon* encodes a protein belonging to subclass III of SnRK2 family, exhibits higher expression in leaf tissues and responses to both drought stress and ABA treatment

**Fig. 2:**
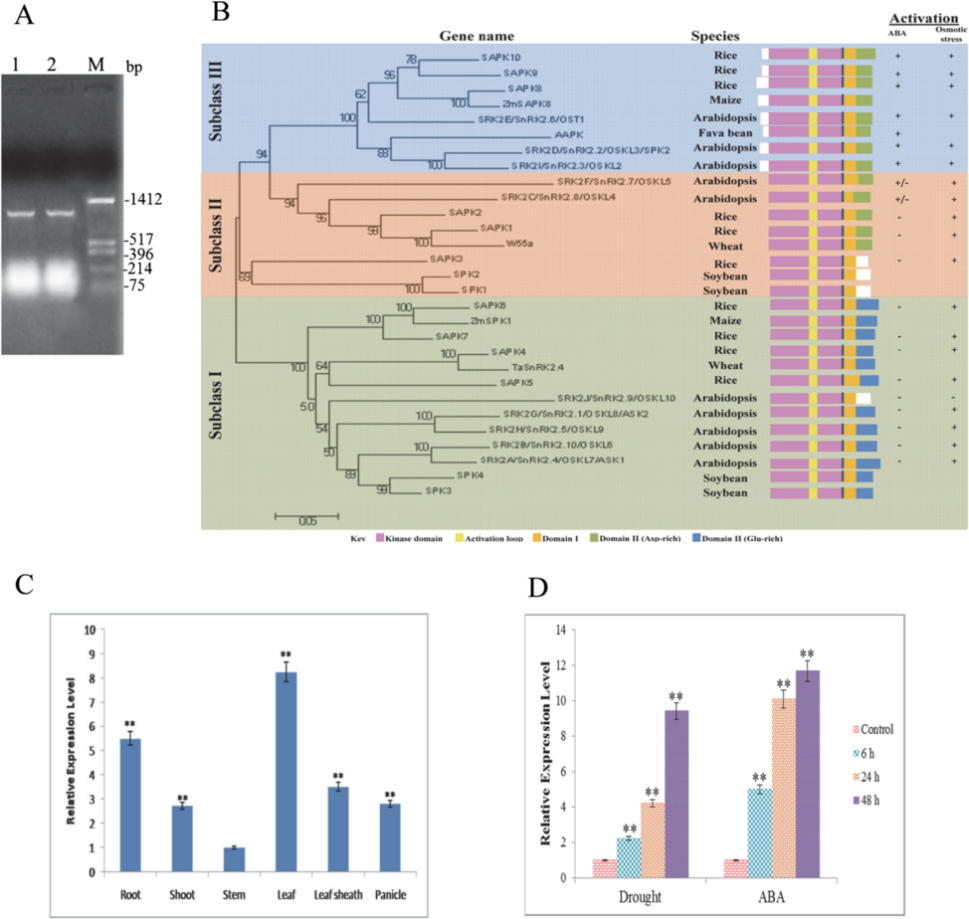
Analyses of phylogenetic relationship and tissue-specific expression of *SAPK9* gene cloned from the wild rice *O. rufipogon*. **a** Agarose gel showing the RT-PCR amplification of *SAPK9* coding DNA sequence (CDS) from drought-tolerant *O. rufipogon*. Lane 1-2 PCR product, Lane M- molecular marker. **b** Phylogenetic tree of SnRK2 family proteins from selected plant species. The phylogenetic tree was constructed in MEGA6.0 software with the neighbor-joining method. The numbers indicate the bootstrap values (1000 replications). **c** Analysis of real-time PCR depicting *SAPK9* transcript expression in root, shoot, leaf, leaf sheath and panicle in *O. Rufipogon*. **d** Real-time PCR analysis of the SAPK9 transcript in leaf samples of *O. rufipogon* under dehydration and exogenous ABA (100 μM) treatment at 6, 24 and 48 h. Untreated 0 h sample in each cases were used as control. For internal reference, rice *OsUbi1* gene was used. Error bars represent the mean ± SD of triplicate measurements. Student’s *t*-test was performed to find out statistically significant differences (**P < 0.01)

The full-length CDS of *SAPK9* gene was cloned after isolation from the drought-tolerant rice genotype *O. rufipogon* by RT-PCR (Fig. 2a). Comparison of the cloned gene sequence (accession no. KT387673) with a previously reported sequence (accession no. AB125310) of *SAPK9* gene from a *japonica* rice cultivar revealed alternation of the nucleotide at two positions. A nucleotide modification (C to T) at the 746^th^ position from the start codon created the 249^th^ amino acid change from proline (P) to leucine (L); and another nucleotide modification (A to C) at the 970^th^ position from the start codon altered the 324^th^ amino acid from serine (S) to arginine (R) (Additional file 5: Figure S3). The phylogenetic analysis of rice SnRK2 family members (designated as SAPKs) with *Arabidopsis* SnRK2s and related proteins from other plants revealed that the ten SAPK proteins of rice are clustered into three subclasses (Fig. 2b). Each subclass contains members from both *Arabidopsis* and rice SnRK2, demonstrating that the distinction of these subclasses originated before the divergence of dicots and monocots. The SAPK9 was found to be clustered into subclass III of SnRK2 family along with the well characterized ABA-activated SnRK2 genes, i.e. AAPK of *Vicia faba* [5] and OST1 (SnRK2.6/SRK2E) of *Arabidopsis* [6, 7], which have been reported to be involved in the ABA-regulated stomatal closure. Apart from these two genes, the SAPK9 was observed to be grouped with SnRK2.2 and SnRK2.3 of *Arabidopsis* [41], and SAPK8 and SAPK10 of rice [23] in the subclass III of SnRK2 family. Growing evidence indicate that subclass III SnRK2s are the global regulators of multiple stress signaling pathways, and these proteins have certain characteristic functional domains (Fig. 1d).

The tissue specificity of *SAPK9* gene was monitored through real-time PCR analyses using the total RNA sample isolated from different tissues, namely root, shoot, stem, leaf, leaf sheath and panicle. The strongest expression of *SAPK9* transcript was found in the leaf followed by root (Fig. 2c). Further, to analyse the transcriptional responses of *SAPK9* gene under drought stress and ABA treatment, real-time PCR analysis was performed. The *SAPK9* expression was observed to be significantly induced by exogenous ABA within 6 h of treatment in comparison to drought stress. However, the *SAPK9* expression upon drought stress increased significantly after 24 h (Fig. 2d). The results indicate that the *SAPK9* is involved in responses to both drought stress and ABA treatment, confirming the subclass III nature of this SnRK2 member [23].

### The SAPK9 protein has a characteristic kinase fold structure and the recombinant protein possesses autophosphorylation and transphosphorylation activities

**Fig. 3:**
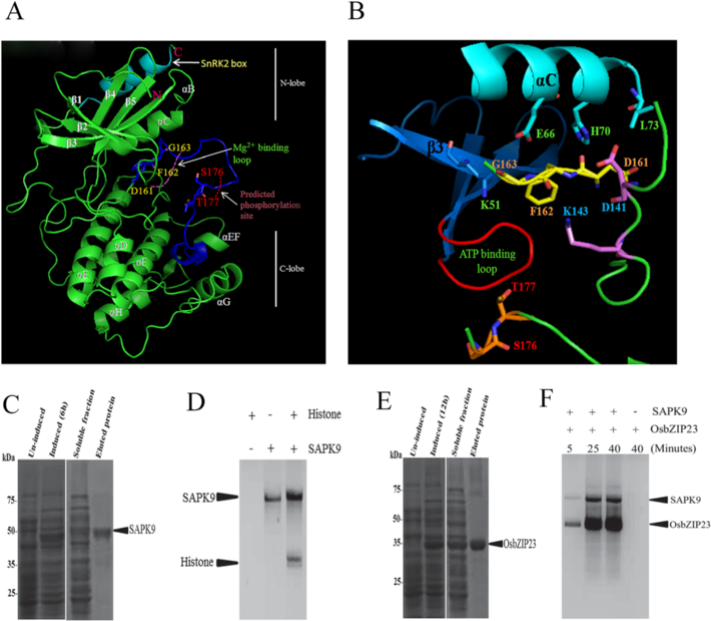
*In silico* homology model of SAPK9 protein and kinase activity of the recombinant SAPK9 on different substrates. **a** The model of SAPK9 was constructed by Modeller9.15 (https://salilab.org/modeller/9.15) using *Arabidopsis* SnRK2.6 (PDB ID: 3UC4) as a template. The SnRK2 box and the activation loop segment are highlighted in cyan and blue, respectively. Predicted phosphorylation sites in activation loop are marked in red (S176, T177) and the Mg^2+^ binding loop is indicated. **b** Close view of the catalytic domain, ATP binding loop and phosphorylation site of SAPK9 activation loop. **c** Coomassie blue stained SDS-PAGE showing *E. coli* expressed recombinant His-tagged SAPK9 protein purified through Ni-NTA chromatography under native condition. **d** Autoradiography showing in vitro phosphorylation of histone III substrate with recombinant SAPK9. **e** Coomassie blue stained SDS-PAGE showing *E.coli* expressed recombinant His-tagged OsbZIP23 protein purified through Ni-NTA chromatography under native condition. **f** Autoradiography showing in vitro phosphorylation of OsbZIP23 substrate with recombinant SAPK9

Analysis of the amino acid sequence (362 amino acids) derived from the cloned *SAPK9* CDS revealed that the predicted molecular mass of SAPK9 is ∼ 40 kDa. The SAPK9 shares 80 and 76 % sequence similarity with the two reported Snf1-related kinase proteins viz., SnRK2.6 and SnRK2.3 of *Arabidopsis thaliana*, respectively (Additional file 6: Figure S4). Using *Arabidopsis* SnRK2.6 (PDB ID: 3UC4) as a template, the homology modelling revealed the bilobal (N-lobe and C-lobe) kinase fold structure of SAPK9 (Fig. 3a). The quality of the model displayed 94.5 % residues in the core region and 2.5 % residues in the allowed region. Interestingly, the *in silico* structure of catalytic SAPK9 displayed the canonical Ser/Thr kinase fold, identical to other Snf1 kinase domains. The well-ordered characteristic SnRK2 box (Fig. 3a) forming a single α-helix was found to be present in the N-terminal lobe packed parallel against the αC helix. In the N-terminal lobe, five β-sheets plus one α-helix called helix αC was found. The C-terminal lobe was found to be larger and mainly helical (Fig. 3a). The activation loop was found to be present at amino acid position 161 to 187. In the activation loop, S176 and T177 were predicted to be the phosphorylation site for SAPK9 activation (Fig. 3a). The catalytic cleft resides at the junction of the larger C-terminal lobe, and this cleft contains the substrate-binding site, the ATP binding G-loop and the Mg^2+^ binding site (Fig. 3b). It has been established before that the αC-helix in the N-terminal lobe is required for correct folding of the catalytic centre and kinase activation loop, and it is stabilized by the SnRK2 box (Fig. 3a and b) [42].

To study the kinase activity of recombinant SAPK9 in vitro, the bacterially expressed 6xHis-tagged SAPK9 protein was purified by Ni-NTA chromatography under native condition (Fig. 3c) and kinase assay was performed. It was observed that the generic substrate histone III was phosphorylated by the recombinant kinase (Fig. 3d). Moreover, the occurrence of an extra band corresponding to the size of recombinant SAPK9 (~42 kDa) suggested that it is able to autophosphorylate. This was confirmed by performing the kinase assay in the absence of substrate (Fig. 3d). These results are in accordance with the previous reports on the kinase activity of subclass III SnRK2s, OST1/SnRK2.6 and ZmSnRK2.8 [43, 44]. It is known that the AREB/ABF proteins require the phosphorylation of their multiple conserved sites by SnRK2 protein kinases for ABA-dependent activation [21, 41, 45]. Previous studies have shown that the SAPK9 can phosphorylate the downstream AREB/ABF type transcription factors such as TRAB1 and OsbZIP46 [46, 47]. As OsbZIP23 is phylogenetically very close to OsbZIP46, therefore we investigated the potential phosphorylation activity of SAPK9 on OsbZIP23 substrate. For this, the N-terminal 6xHis-tagged OsbZIP23 was expressed in the bacterial system and the recombinant OsbZIP23 protein was purified by Ni-NTA chromatography under native condition (Fig. 3e) and subsequently, kinase assay was performed for different time intervals. It was found that the SAPK9 phosphorylates OsbZIP23, and the phosphorylation efficiency is increased with the increase in reaction time (Fig. 3f). These results suggest that the SAPK9 protein has both autophosphorylation and transphosphorylation activities in vitro, and has the potentiality of transactivation of the OsbZIP transcription factors in vivo.

### Generation of transgenic rice lines for SAPK9 overexpression and endogenous gene silencing

**Fig. 4:**
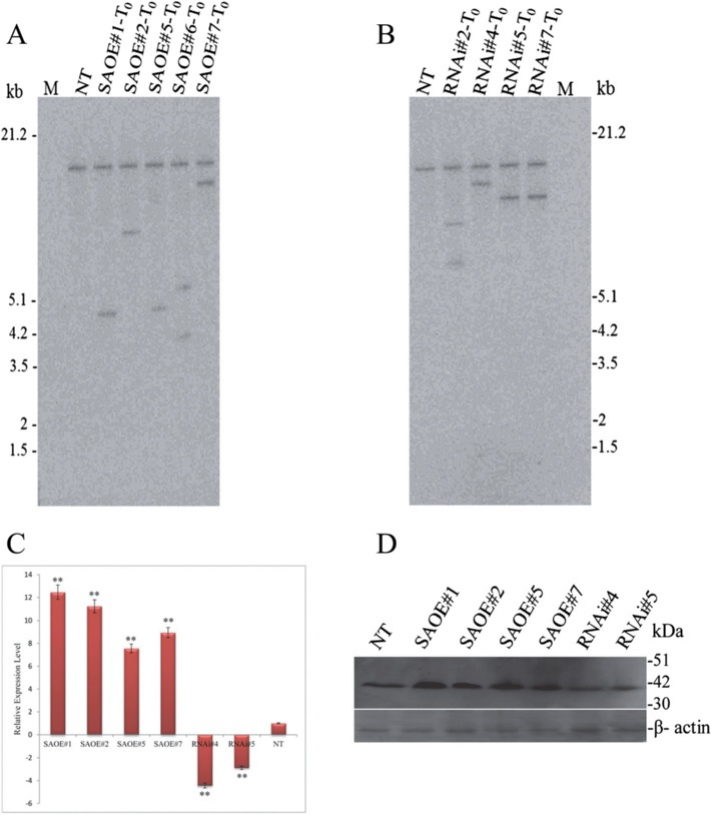
Molecular analyses of the transgenic rice lines developed for SAPK9 overexpression (OE) and RNAi-mediated endogenous gene silencing (RNAi). **a** Southern hybridization blot of T_0_ transformants of OE plants (designated as SAOE#1, 2, 5, 6 and 7). **b** Southern hybridization blot of T_0_ transformants of RNAi plants (designated as RNAi#2, 4, 5 and 7). For both (**a**) and (**b**), the *Hind*III-digested genomic DNA samples were used to probe with the 470 bp fragment of *SAPK9* CDS. Lane NT- non-transgenic control, Lane M- molecular weight marker. **c** The relative expression level of *SAPK9* gene was analysed in leaf tissues of OE, RNAi, and NT plants in vegetative stage through real-time PCR. For internal reference, the *OsUbi1* gene was used. Error bars represent the mean ± SD of triplicate measurements. Student’s *t*-test was performed to find out statistically significant differences (**P < 0.01). **d** Western blot illustrating the expression level of SAPK9 protein (upper panel) in leaf tissues of OE, RNAi and NT plants, where β-actin protein (lower panel) showing equal loading in each lane

To investigate the *gain-of-function* and *loss-of-function* phenotypes of *SAPK9* gene in rice through transgenesis*,* gene overexpression (OE) and RNAi-mediated gene silencing (RNAi) constructs were prepared using *OsUbi1* promoter and nopaline synthase (NOS) transcription terminator in the pCAMBIA1301 binary plasmid (Additional file 2: Figure S5A and B). Both the constructs were then separately incorporated into drought-sensitive *indica* rice cultivar IR20 by *Agrobacterium*-mediated transformation. The putative OE and RNAi transformants were subjected to hygromycin selection during in vitro culture. The hygromycin selected transformants were verified by means of Southern hybridization using *SAPK9* gene-specific probe to detect transgene integration patterns in T_0_ plants. Southern blot analysis detected single and multiple integration lines in both OE and RNAi (Fig. 4a and b). The OE lines of *SAPK9* were designated as SAOE#1, 2, 3 etc., and RNAi lines were designated as RNAi#1, 2, 3 etc. The single integration T_1_ plants of OE and RNAi lines were examined for the expression level of *SAPK9* transcript by real-time PCR. The real-time PCR analysis revealed that the relative expression level of *SAPK9* transcript increased significantly (*P* < 0.01) in OE lines, but decreased significantly (*P* < 0.01) in RNAi lines compared to non-transgenic (NT) plants (Fig. 4c). Similarly, western blot analysis disclosed the increased expression of SAPK9 protein in OE lines in comparison to RNAi lines and NT plants (Fig. 4d), corroborating the real-time PCR result. Further, we have also checked the expression of *SAPK9* gene at the reproductive stage of the transgenic and NT plants (Additional file 7: Figure S7), and the analysis revealed the similar level and trend of expression as observed in the vegetative stage. The high sequence similarity of SAPK9 with the other two members of SnRK2 subclass III in rice viz., SAPK8 and SAPK10, led us to analyse the relative expression levels of these two genes in transgenic lines and NT plants. It was observed that in OE and RNAi lines the relative expression levels of both the genes were not much altered in comparison to NT plants (Additional file 8: Figure S6).

### Overexpression of *SAPK9* improves drought tolerance in rice by increasing the water retention capacity in transgenic plants through osmotic adjustment and stomatal closure

**Fig. 5:**
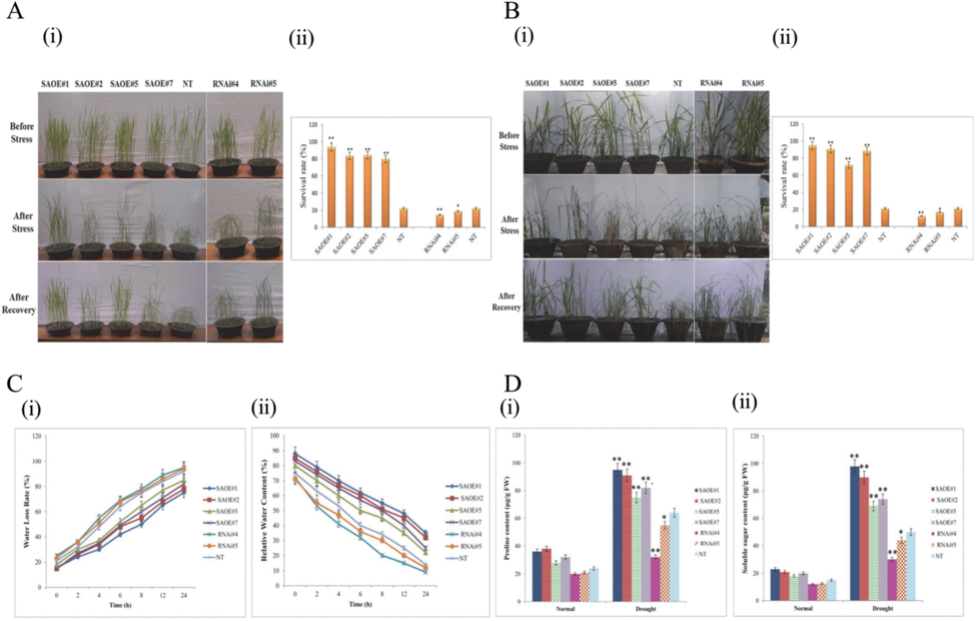
Evaluating drought stress tolerance of *SAPK9* overexpressed (OE) and gene silenced (RNAi) transgenic rice plants. **a**-i Pictures showing three sets of plants, i.e. OE, RNAi and NT in the vegetative stage under the conditions of before drought stress, after drought stress and subsequent recovery after drought stress. **a-**ii Survival rates (%) of OE, RNAi, and NT plants in the vegetative stage. **b**-i Pictures showing OE, RNAi, and NT plants in early reproductive (panicle initiation) stage under the conditions of before drought stress, after drought stress and subsequent recovery after drought stress. **b**-ii Survival rates (%) of OE, RNAi and NT plants in the early reproductive stage. **c**-i Comparison of water loss rate (WLR) and (**c**-ii) relative water content (RWC) of the detached leaves from transgenic lines and NT plants at the five-leaf stage. Estimation of the contents of (**d**-i) proline and (**d**-ii) soluble sugar in leaf tissues of transgenic lines and NT plants before and after drought stress

To examine the drought stress tolerance capacity of SAPK9 OE and RNAi lines at the vegetative and early reproductive (panicle initiation) stages, hygromycin selected T_1_ plants were transferred into the pots and allowed to grow under glasshouse conditions for 30 days along with non-transgenic (NT) plants. After one week of transferring the pots in net-house, each set of plants from vegetative and panicle initiation stage were subjected to drought stress by withholding water supply until the visible effect of dehydration was noticed in RNAi lines and NT plants. After the duration of drought stress, plants were watered for 3 days in each case, and the survival rate (%) was determined. A significantly (*P* < 0.01) greater survival rate was observed in OE lines when compared with RNAi lines and NT plants during both vegetative stage (Fig. 5a) and reproductive stage (Fig. 5b). The observed increase and decrease in the drought tolerance ability of OE and RNAi lines, respectively, clearly indicated that the SAPK9 plays a strong role in drought tolerance in rice. Previous studies on either overexpression or silencing of the ABA-responsive kinase genes in rice and other plant species have documented similar results of drought tolerance or drought sensitivity, respectively [5, 9, 16, 31, 48].

Globally, water availability dictates the yield of crop plants including rice [34]. Plant with high water holding capacity can sustain the severity of drought stress better than the others. The water status in a plant body is principally determined by two critical parameters- water loss rate (WLR) and relative water content (RWC) [31, 49]. The WLR and RWC were measured in leaf tissues of OE, RNAi lines, and NT plants. It was observed that the OE lines exhibited lower WLR and higher RWC in contrast to the RNAi lines, which showed higher WLR and lower RWC in comparison with NT plants (Fig. 5c). These results indicate that the SAPK9 plays an important functional role in enhancing the water retention capacity of the plants under water deficit condition.

**Fig. 6:**
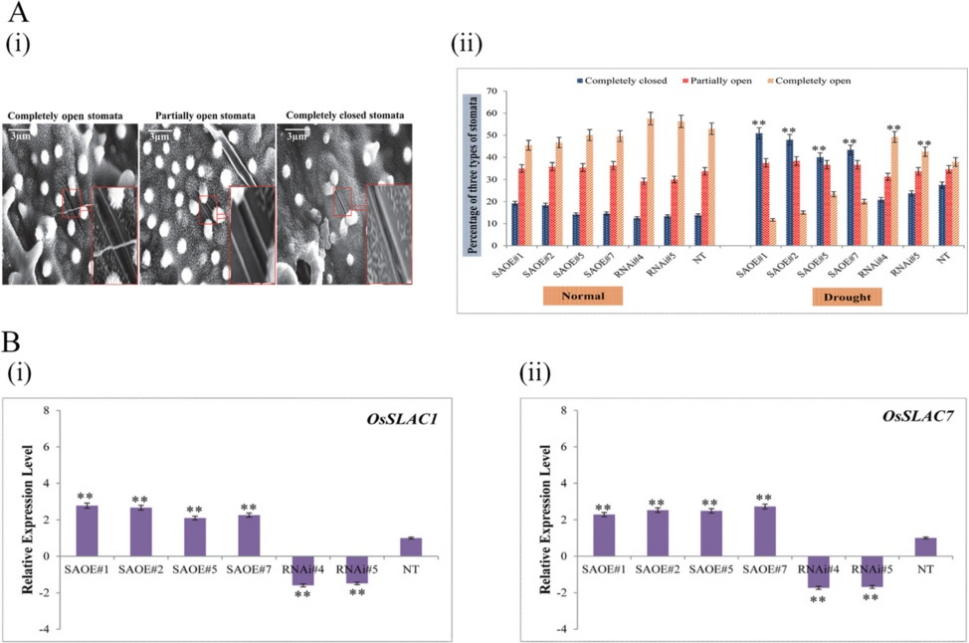
Monitoring stomatal closure and relative expression level of stomatal genes in SAPK9 overexpressed (OE) and gene silenced (RNAi) transgenic rice plants. **a**-i Representative photographs showing stomatal status in rice leaves. **a**-ii Number of completely open, partially open and completely closed stomata were calculated before (Normal) and after stress (Drought) in three sets of plants, i.e. OE, RNAi and NT plants (*n* = 80). All the results were compiled from three independent experimental sets. Error bars represent the mean ± SD of triplicate measurements. Student’s *t*-test was performed to find out statistically significant differences (**P < 0.01). **b** Real-time PCR analysis showing transcript level of stomatal genes- (i) *OsSLAC1* and (ii) *OsSLAC7* in transgenic and NT plants during drought stress. For internal reference, the *OsUbi1* gene was used. Error bars represent the mean ± SD of triplicate measurements. Student’s *t*-test was performed to find out statistically significant differences (**P < 0.01)

Additionally, we investigated the mechanism by which the SAPK9 endows the OE rice plant for its improved water holding capacity. The two major physiological mechanisms that reduce water loss in plants under drought stress condition are the osmotic adjustment and stomatal closure. It has been established that plants under drought stress condition accumulate compatible osmolytes such as free proline and soluble sugars in order to keep the cellular structure intact by adjusting the intracellular osmotic potential [34]. The physiological mechanism by which the overexpression of SAPK9 improves the water retention capacity of transgenic rice lines under drought stress was elucidated by determining the contents of proline and soluble sugar in the transgenic and NT plants. Analysis of the experimental data revealed not much difference in proline and soluble sugar contents of the OE, RNAi lines and NT plants while growing them under normal condition. However, upon drought stress imposition, higher contents of proline and soluble sugar were accumulated in the OE lines compared with RNAi and NT plants (Fig. 5d). The significant increase in the cellular osmolyte contents in OE lines during drought stress strongly suggests that the accumulation of these osmolytes occurs due to the enhanced activity of SAPK9, the possible molecular mechanism of which has been discussed in the later section. On the contrary, the reduced activity of SAPK9 in RNAi lines results in decreased accumulation of osmolytes, consequently leading to drought sensitivity. Similar phenotypic observations associated with cellular osmolyte concentration have been documented previously upon up-regulation or down-regulation of candidate genes [25, 31, 50, 51]. It is to be noted that in our study the analysis has been performed after a drought stress treatment over a period of 8 days under net-house condition (when the clear phenotypic difference was observed among OE, RNAi and NT plants). However, for a comprehensive physiological evaluation of OE and RNAi lines to compare with the NT plants, the analyses need to be carried out over a longer duration in the field condition, as has been suggested before [52]. Previous reports have shown that the members of subclass III SnRK2, i.e., *Arabidopsis* OST1/SnRK2.6 and fava bean AAPK are involved in the ABA-regulated stomatal movement [5–7]. Hence in the present study, the stomatal status was observed and counted in OE lines, RNAi lines, and NT plants. Under normal growth conditions, the differences in the stomatal status of transgenic lines and NT plants were minimal. After the drought stress treatment, ~20–23 and ~27 % of stomata were completely closed in RNAi and NT plants, respectively; while greater proportions (~40–50 %) of closed stomata were present in OE lines (Fig. 6a). Accordingly, the proportions of completely opened stomata were significantly lowest in OE lines among the three sets of plants (Fig. 6a). Interestingly, the proportions of partially opened stomata in OE lines were comparatively highest. The variation in the stomatal movement of transgenic lines and NT plants led us to verify the expression level of two slow anion channel-associated (SLAC) genes viz., *OsSLAC1* and *OsSLAC7*. The results showed that the transcript expression levels of both the genes were significantly (*P* < 0.01) higher in OE lines, compared with the RNAi lines and NT plants (Fig. 6b). Recent investigations have documented that these two stomatal proteins are involved in anion transport [53, 54], and the SAPK8 has been found to activate the function of OsSLAC1 [53]. Our results suggest that the stomatal closure is positively regulated by the functional activity of SAPK9 upon *OsSLAC1* and *OsSLAC7* gene expression. It is anticipated that the phosphorylation ability of SAPK9 might activate the transcription factor(s) specifically required for the expression of these two genes. However, the detailed molecular mechanism of SAPK9-regulated stomatal closure requires further study.

### Transgenic rice lines overexpressing SAPK9 show reduced lipid peroxidation and increased antioxidant activity

**Fig. 7:**
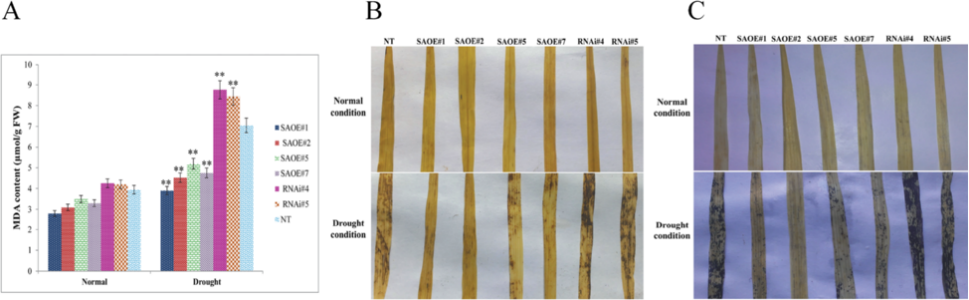
Analysis of malondialdehyde (MDA) content and reactive oxygen species (ROS) activity in *SAPK9* OE and RNAi plants. **a** Estimation of MDA content in leaf tissues of transgenic and NT plants before (Normal) and after drought stress (Drought). Error bars represent the mean ± SD of triplicate measurements. Student’s *t*-test was performed to find out statistically significant differences (**P < 0.01). **b** Monitoring ROS activity, as revealed by evaluation of H_2_O_2_ content in leaf tissues of transgenic and NT plants through visualization upon staining with DAB under normal and drought conditions. **c** Accumulation of O^2-^ ions in leaf tissues was visualized by staining with NBT in transgenic and NT plants under normal and drought stress conditions. Results were recorded from three independent experimental sets, and the result from one set is documented here

Stresses usually cause harm in plants via oxidative damage by generating reactive oxygen species (ROS), such as H_2_O_2_ and O^2-^ ions. The ROS are toxic and highly reactive oxygen derivatives that damage macromolecules such as DNA, protein and carbohydrate, resulting in cell death [48]. ROS causes lipid peroxidation, which results in the production of malondialdehyde (MDA) [55]. The MDA is a stress-specific molecular marker that measures the extent of cellular damage due to the imposition stress in plants. Since the overexpression of SAPK9 improved drought tolerance in rice plants through osmotic adjustment and stomatal closure, hence we examined whether the SAPK9 functions in stress tolerance through ROS detoxification. The content of MDA in OE lines, RNAi lines, and NT plants was measured and analysed. There were marginal variations in the content of MDA in OE, RNAi lines, and NT plants during normal growth condition. However, under drought stress, the MDA content in OE lines was significantly (*P* < 0.01) lowest among the three sets of plants, although the lipid peroxidation levels increased in all plants compared to the normal condition (Fig. 7a). The reduced MDA level under drought stress in the OE lines implied that the cellular oxidative damage is less severe in these lines in comparison with RNAi lines and NT plants. Therefore, we were interested in documenting the ROS accumulation by staining the leaves of OE, RNAi lines, and NT plants with 3,3′-diaminobenzidine (DAB) and nitro blue tetrazolium (NBT). Under normal growth conditions, there were no differences in the localized peroxide and free radicals in leaf tissues amongst transgenic lines and NT plants. However, upon exposure to drought condition, the leaf tissues of RNAi and NT plant displayed higher levels of H_2_O_2_ and O^2-^ accumulation in comparison with the OE lines (Fig. 7b and c). These results confirm that the SAPK9 overexpression *in planta* has enhanced the drought tolerance phenotype of OE plants by ROS detoxification, which resulted in less membrane damage.

### Overexpression of SAPK9 causes elevated transcript expression of ABA- and stress-responsive genes resulting in increased ABA sensitivity at germination and post-germination stages of transgenic rice

**Fig. 8:**
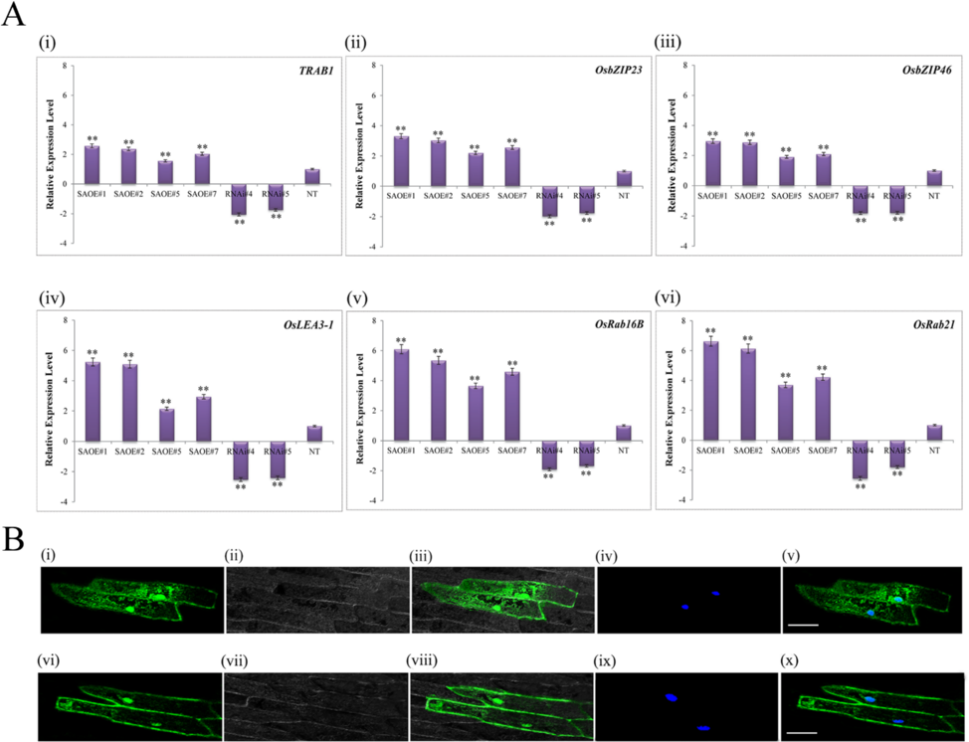
Expression profiling of a few drought stress inducible genes working hierarchically downstream of the ABA-signaling pathway and analysis of subcellular localization of SAPK9 protein in onion epidermal cells. **a**-i-vi Analysis of real-time PCR depicting transcript level of *TRAB1*, *OsbZIP23*, *OsbZIP46*, *OsLEA3*-1, *OsRab16B* and *OsRab21* in leaf tissues of transgenic and NT plants during drought stress. For internal reference, the *OsUbi1* gene was used. Error bars represent the mean ± SD of triplicate measurements. Student’s *t*-test was performed which indicated statistically significant differences (**P < 0.01). **b** SAPK9–GFP fusion protein and control GFP were transiently expressed in onion epidermal cells and observed with a laser-scanning confocal microscope. Upper panel (i-v): SAPK9–GFP fusion; lower panel (vi-x): only GFP. Images were taken (i, vi) in the dark field for green fluorescence, (ii, vii) in the bright field, (iii, viii) dark field green fluorescence merged with bright field image and (iv, ix) DAPI stained nucleus (5 μg/mL) (v, x) DAPI stained nucleus merged with dark field green fluorescence. Scale bar- 100 μm

To understand the comprehensive function of *SAPK9* under drought stress condition, we analysed the expression profiles of some drought-inducible genes, such as *TRAB1*, *OsbZIP23*, *OsbZIP46*, *OsLEA3-1*, *OsRab16B*, and *OsRab21*, which work hierarchically downstream of *SAPK9* in the ABA-signaling pathway. The *TRAB1*, *OsbZIP23*, and *OsbZIP46* are the rice bZIP transcription factors, known to be induced by ABA and drought stress [25, 47, 56, 57]. Two dehydrin proteins are encoded by *OsRab16B* and *OsRab21*, which are transcriptionally responsive to abiotic stress [58, 59]. OsLEA3-1 is a late embryogenesis abundant (LEA) protein proposed to protect cell membrane structure stable by minimizing the damage caused by increasing ion concentration in dehydrating cells [60] and improves drought tolerance [61]. We found that the relative expression level of *TRAB1*, *OsbZIP23*, *OsbZIP46*, *OsLEA3-1*, *OsRab16B*, and *OsRab21* increased significantly (*P* < 0.01) in OE lines, compared with the NT plants (Fig. 8a). However, the levels of expression of these genes in RNAi lines were significantly (*P* < 0.01) lower in comparison with NT plants (Fig. 8a). These results suggest that the SAPK9 might be a transcriptional activator of these drought-stress associated genes in the ABA signaling pathway in rice, and overexpression or downregulation of *SAPK9* alters the expression of these set of genes. Since most of the transcriptional activator proteins are usually localized in the nucleus, therefore we examined the subcellular localization of SAPK9 protein in onion epidermal cells. We found increased fluorescence in cytoplasm and nucleus due the expression of SAPK9–GFP fusion protein compared to that of only GFP, indicating the SAPK9 protein was localized in these cellular compartments (Fig. 8b). The subcellular localization of SAPK9 is similar to the previous reports on other SnRK2 family members [16–18]. The exact molecular mechanism of SAPK9-mediated transcriptional activation is yet to be investigated.

**Fig. 9:**
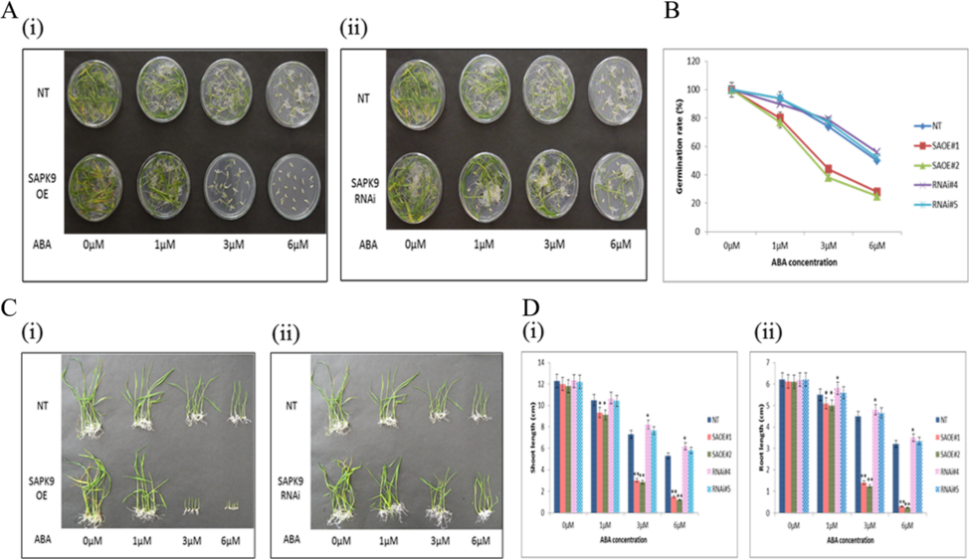
Assessment of ABA sensitivity of *SAPK9* OE and RNAi plants at seed germination and post-germination stages. **a**-i, ii Germination performance of seeds grown on MS agar media supplemented with 0, 1, 3 and 6 μM ABA from two OE lines (SAOE#1, SAOE#2), and two RNAi lines (RNAi#4, RNAi#5) compared to NT plants at 10^th^ day. **b** Germination rate (%) of seeds grown on media supplemented with 0, 1, 3 and 6 μM ABA from OE, RNAi and NT plants at 10^th^ day. **c**-i, ii The performance of transgenic and NT seedlings grown in ½ MS liquid media supplemented with 0, 1, 3 and 6 μM of ABA after 14 days. **d** Comparison of (i) shoot length and (ii) root length of transgenic and NT seedlings grown in media supplemented with 0, 1, 3 and 6 μM ABA after 14 days. For representation and better comparison amongst OE, RNAi and NT plants, the NT panel is duplicated in A (i and ii) and C (i and ii). Error bars represent the mean ± SD of triplicate measurements. Student’s *t*-test was performed to find out statistically significant differences (*P < 0.05, ** *P* < 0.01). Results were compiled from three independent experimental sets

The role of phytohormone ABA in the maintenance of seed dormancy, hindrance of seed germination and inhibition of seedling growth is well documented [62]. Since the expression of *SAPK9* gene is profoundly induced by ABA (Fig. 2d), and ABA-responsive gene expressions are increased in OE lines (Fig. 8a); hence we have investigated the ABA sensitivity of transgenic lines at germination and post-germination stages. The seeds from two independent OE lines (SAOE#1 and SAOE#2), two RNAi lines (RNAi#4 and RNAi#5) and non-transgenic (NT) plants were germinated on MS agar media with variable concentrations of ABA (0, 1, 3 and 6 μM). There were no significant differences in the germination rate of OE lines at 0 and 1 μM ABA, but at 3 and 6 μM ABA concentration germination rate of OE lines was significantly lowest in comparison with RNAi lines and NT plants (Fig. 9a and b), implying that the *SAPK9* OE lines are most sensitive to ABA at the germination stage. We also investigated the ABA sensitivity of OE and RNAi lines at the post-germination stage. The results showed no significant differences in the shoots and roots length of OE, RNAi and NT seedlings grown at 0 μM concentration of ABA for 14 days. However, at 1, 3 and 6 μM ABA concentrations, the length of shoots and roots of OE lines decreased significantly (*P* < 0.01) in comparison with RNAi lines and NT plants (Fig. 9c and d). These findings indicate that the overexpression of *SAPK9* increases ABA sensitivity at the post-germination stage as well. Together with the finding on decreased ABA sensitivity of SAPK9 down-regulated RNAi lines*,* we infer that the *SAPK9* is a positive regulator of ABA-dependent stress signaling pathway in rice. Our observations are consistent with the previous reports that overexpression and silencing of ABA-responsive gene results in differential ABA sensitivity at both germination and post-germination stages in rice [25, 31, 63].

### Overexpression of SAPK9 increases grain yield in transgenic rice under drought stress by improving pollen maturation, spikelet fertility, and panicle weight

**Fig. 10:**
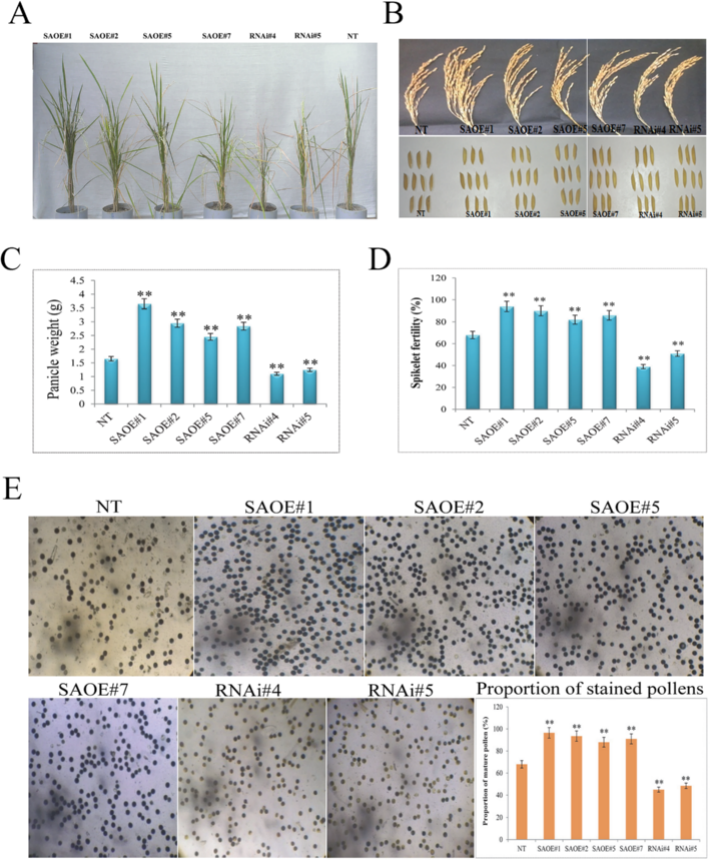
Evaluating grain yield of *SAPK9* overexpressed (OE) and gene silenced (RNAi) transgenic rice plants. **a** Drought stress treatment of three sets of plants, i.e. OE, RNAi and NT plants at flowering stage in PVC pipes and subsequent irrigation till seed maturation stage. **b** Mature panicles and grains of three sets of plants. **c** Comparison of panicle weight in three sets of plants. **d** Comparison of spikelet fertility (%) in three sets of plants. **e** Proportion (%) of viable pollens (after staining with 1 % I_2_-KI solution) in three sets of plants. Error bars represent the mean ± SD of triplicate measurements. Student’s *t*-test was performed to find out statistically significant differences (** *P* < 0.01). Results were compiled from three independent experimental sets

Since there was a striking difference between the transgenic OE and RNAi plants with respect to the duration of drought tolerance, therefore we were encouraged to examine the grain yield attributing traits of these plants grown under drought stress. For this, two sets of OE lines, RNAi lines and NT plants were grown in PVC pipes under glasshouse condition till they reach the panicle imitation stage (~60 days of seeding), and subsequently transferred to net-house. Upon attaining the flowering stage, plants were subjected to drought stress condition for a period of 10 days. After completion of the stress period plants were watered normally, and allowed to recover through the flowering stage until the seed maturation occurs (Fig. 10a). Under drought stress condition, the panicle weight and spikelet fertility of OE lines were found to be significantly (*P* < 0.01) highest among the three sets of plants (Fig. 10b, c and d). There was no obvious difference in grain length and width amongst transgenic lines and NT plants (Fig. 10b). These results indicate that the *SAPK9* gene plays a significant role in increasing the grain yield in rice by influencing panicle weight and spikelet fertility. Since panicle weight and spikelet fertility are correlated with the pollen maturation and viability, therefore the number of matured and viable pollens in OE, RNAi, and NT plants were investigated using I_2_-KI staining. The analysis revealed that OE lines had a higher proportion of mature pollen than those of RNAi lines and NT plants (Fig. 10e). The proportion of mature pollen reflects the pollen viability. The more number of mature pollens correlating with the overexpression of *SAPK9* suggested that the SAPK9 functions in increasing pollen viability. Additionally, we have already found that the relative expression level of *SAPK9* is significantly high in panicle under drought stress condition (Fig. 2c). Collectively, these results document that the SAPK9 enhances spikelet fertility by regulating pollen maturation and thereby improves grain yield. Interestingly, previous reports have documented that the SAPK9 plays some functional role in the development of panicle [64] as well in the pollen [65] via ABA-signaling pathway. However, the exact molecular mechanism by which the SAPK9 gene regulates the spikelet fertility and grain yield in rice remain to be elucidated.

### Possible molecular mechanism of SAPK9 action

Based on the findings of the present study and the information available in the literature, a molecular interactive pathway for SAPK9 has been proposed (Additional file 9: Figure S8). Our work clearly established that the SAPK9 acts as a transactivating kinase and a potential transcriptional activator for modulating the activity of several key components in the ABA-dependent stress adaptation pathway in rice plant. We hypothesize that an unknown factor(s) might be involved in mediating the transcriptional activity of SAPK9, which requires further investigation.

## Conclusion

We have characterized the structural, biochemical and physiological functions of SAPK9, a subclass III SnRK2 gene of rice through developing *gain-of-function* and *loss-of-function* mutants by transgenesis. The *SAPK9* transcript expression has been found to be differentially regulated in the selected drought-tolerant and drought-sensitive rice genotypes, and its expression is comparatively more elevated in reproductive stage than the vegetative stage. The *SAPK9* transcript has been observed to be upregulated by drought stress and ABA treatments. The SAPK9 protein has characteristic kinase fold structure, and is able to transactivate its target substrates, including histone III and OsbZIP23; and itself is autophosphorylated. Constitutive overexpression of *SAPK9* gene from a drought-tolerant wild rice genotype and RNAi-mediated silencing of endogenous *SAPK9* gene in a drought-sensitive cultivar have revealed that the SAPK9 positively regulates drought stress tolerance by boosting the osmotic adjustment and stomatal closure of the plant. The enhanced drought-tolerant transgenic rice lines overexpressing SAPK9 display less cellular oxidative damage resulting from the reduced reactive oxygen species accumulation. The SAPK9 overexpressed lines exhibit increased sensitivity to exogenous ABA and enhanced transcription of other hierarchically downstream ABA-responsive genes, indicating that the SAPK9, indeed, is a positive regulator of ABA-mediated stress signaling pathway in rice plant. The increased functional activity of SAPK9 overexpression in transgenic lines improves grain yield-related traits such as panicle weight and spikelet fertility by increasing the pollen viability. Together, the present findings strengthen our knowledge about the functional role of SAPK9 as transactivating kinase and a probable transcriptional activator, which can be utilized as a promising gene-based molecular marker in transgenic breeding for generating crop plants with improved drought tolerance and grain yield.

## Abbreviations

ABA, Abscisic acid; CDS, Coding DNA sequence; DAB, 3,3′-diaminobenzidine; MDA, Malondialdehyde; NBT, Nitro blue tetrazolium; NT, Non-transgenic; OE, Overexpression; RNAi, gene silencing by RNA interference; ROS, Reactive oxygen species; RWC, Relative water content; SAPK, Osmotic stress/ABA-activated protein kinase; SLAC, Slow anion channel-associated; SNP, Single nucleotide polymorphism; SnRK2, Sucrose non-fermenting 1-related kinase 2; WLR, Water loss rate.

## Additional files

Additional file 1: Table S1. List of primers used in this study. (DOCX 21 kb) Additional file 2: Figure S5. Schematic representation of the genetic constructs based on pCAMBIA1301 plasmid used for *Agrobacterium*-mediated transformation of drought-sensitive *indica* rice cultivar IR20. (A) The gene overexpression (OE) construct of *SAPK9* carrying 1086 bp CDS from *O. rufipogon*. The developed transgenic rice lines were designated as SAOE#1, 2, 3 etc. (B) The RNAi-mediated gene silencing (RNAi) construct of endogenous *SAPK9* gene. The 605 bp 5′-part of *SAPK9* CDS from *O. rufipogon* was cloned in sense and antisense orientation flanking an arbitrary 200 bp DNA linker. The developed transgenic rice lines were designated as RNAi#1, 2, 3 etc. (TIF 453 kb) Additional file 3: Figure S1. PCR-amplified *SAPK9* CDS from the chosen rice genotypes. Lanes 1-11 represents *O. rufipogon, O. nivara,* Nagina22, Manipuri, Vandana, Swarna, IR20, IR36, IR64, IR72, HRC300, respectively. Lane M- standard molecular weight marker. (TIF 1083 kb) Additional file 4: Figure S2. Multiple sequence alignment for analysis of amino acid polymorphism in the *SAPK9* CDS from the selected rice genotypes using Jalview software. (TIF 3333 kb) Additional file 5: Figure S3. Amino acid sequence alignment of the isolated SAPK9 CDS from wild rice *Oryza rufipogon* (accession no. KT387673) with the reported sequence of *japonica* rice cultivar (accession no.AB125310). (TIF 487 kb) Additional file 6: Figure S4. Multiple alignments showing amino acid sequence similarity of SAPK9 with Arabidopsis OST1/SnRK2.6 (PDB ID: 3UC4) and SnRK2.3 (PDB ID: 3UC3). (TIF 644 kb) Additional file 7: Figure S7. The relative expression level of *SAPK9* gene in reproductive stage (panicle initiation) was analysed through real-time PCR from leaf tissues of three sets of plants, i.e. OE, RNAi and NT plants. For internal reference, rice polyubiquitin1 (*OsUbi1*) gene was used. Error bars represent the mean ± SD of triplicate measurements. Student’s *t*-test was performed to find out statistically significant differences (***P* < 0.01). (TIF 240 kb) Additional file 8: Figure S6. Relative expression level of *SAPK8* and *SAPK10* gene in SAPK9 overexpressed (OE) and gene silenced (RNAi) transgenic rice lines. Analysis of real-time PCR depicting transcript level of (A) *SAPK8* and (B) *SAPK10* in leaf tissues of three sets of plants, i.e. OE, RNAi and non-transgenic (NT) plants under drought stress. For internal reference, rice polyubiquitin1 (*OsUbi1*) gene was used. Error bars represent the mean ± SD of triplicate measurements. Student’s *t*-test was performed to find out statistically significant differences (**P* < 0.01). (TIF 339 kb) Additional file 9: Figure S8. Possible molecular mechanism of SAPK9-regulated drought stress tolerance in rice. Upon drought stress, the SAPK9 is activated by ABA signaling pathway, which in turn phosphorylates the bZIP transcription factors activating the expression of downstream genes involved in maintaining the physiological conditions for cellular homeostasis. Similarly, the SAPK9 also transcriptionally activates (might require an unknown factor) for upregulation of several hierarchically downstream genes including the anion channel genes leading to stomatal closure. (TIF 899 kb)
