# Supplementary material for: The sucrose non-fermenting 1-related kinase 2 gene SAPK9 improves drought tolerance and grain yield in rice by modulating cellular osmotic potential, stomatal closure and stress-responsive gene expression
Source: BMC Plant Biol. 2016 Jul 13;16:158. doi: 10.1186/s12870-016-0845-x (PMC4944446; doi:10.1186/s12870-016-0845-x)
Supplement: Additional file 1: Table S1. — List of primers used in this study. (DOCX 21 kb) [file 12870_2016_845_MOESM1_ESM.docx]

| Purpose Primer Orientation Sequence |
| --- |
| Full length CDS SK9F Forward ATGGAGAGGGCGGCGGCGGGGCCGCTGGGGATGGAGATGCCGAT  SK9R Reverse TTACATGGCATATACGATCTCTCCGCTGCTCTCAATGTCAAGGT |
| Bacterial expression rSK9F Forward ATATTCTCGAGATGGAGAGGGCGGCGGCGGGGC  rSK9R Reverse ACGAATTCCATGGCATATACGATCTCTCCGC  rOsbZIP23F Forward ATTGGATCCATGGATTTTCCGGGAGGGAGCG  rOsbZIP23R Reverse TAGAATTCTCACCATGGACCCGTCAGAGTCCT |
| Southern hybridization probe SA9SF Forward AGCGCCGGCCGCTTCAGCGA  SA9SR Reverse ACTAGCTGGGTTGCCAACA |
| Overexpression construct SAPK9F Forward ATATTGTCGACATGGAGAGGGCGGCGGCGGGGC  SAPK9R Reverse TCGGTACCTTACATGGCATATACGATCTCTCCGC |
| RNAi construct SA9Si-F Forward AGTCGACATGGAGATGCCGATAATGCACGACGGT  SA9Si-F1 Forward TAGGTACCATGGAGATGCCGATAATGCACGACGGT  SA9Si-R Reverse ATGGATCCAACATCACGTAGAGCGTCACACCGCAT  LINK-F Forward ATTAGGATCCGCCGAGGCCGCTGCCAAGGAGGCTGCT  LINK-R Reverse ATTAGGATCCGGCCTTGGCAGCGGCCTCCTTGGCAGCA |
| Subcellular localization SK9SL-F Forward ATATAGATCTGAGAGGGCGGCGGCG  SK9SL-R Reverse CCACTAGTCATGGCATATACGATCTCTCC |
| Real-time PCR SAPK9RT-F Forward ATGGCATGGTTAGCAATC  SAPK9RT-R Reverse GGGTGCCTGCTGCTGGAATT  SAPK10RT-F Forward AATCTCTATCCCCGAGATCAGA  SAPK10RT-R Reverse CTGGCTGCTCATCTTGCT  SAPK8RT-F Forward TCGGGGATCCGTCTTTGA  SAPK8RT-R Reverse GCTGCTCATACTATCATCGT  TRAB1RT-F Forward ATATGGATTCAGCGGGCAAGGG  TRAB1RT-R Reverse TCCACCACCTTCTCCACGTTAC  OsbZIP23RT-F Forward GGAGCTGAACGATGAACTCCAG  OsbZIP23RT-R Reverse TCGGCTCATTCTCTCTAGAACCTC  OsbZIP46RT-F Forward GAACACTGACTGGTCCATGCTG  OsbZIP46RT-R Reverse GAGAGAAGCAACTCTGAAGCTGAG  OsRab16B-F Forward CAACAACCACCAGCAGCA  OsRab16B-R Reverse GATCTTGTCCATGAATCCC  OsRab21-F Forward AGCAGCAGCATGCCATG  OsRab21-R Reverse TGGTGCCGGTGGTCAT  OsLEA3-1-F Forward TTCCCACCAGGACCAGGCTA  OsLEA3-1-R Reverse GTCGCCTCCTTGGTATCCT  OsSLAC1-F Forward ATCACCAAGGACAGGCAGAACG  OsSLAC1-R Reverse TGATGTCGTACACCCTCTTGCC  OsSLAC7-F Forward TACGCTTGCAAGGTGGTCTT  OsSLAC7-R Reverse GGCGCGAAGAAGAAGTTGAC  OsUbi1-F Forward TTGAGGTCGAGTCGTCTGAC  OsUbi1-R Reverse GTGGACTCCTTCTGGATGT |

**Supplementary Table S1: List of primers used in this study**
