# Supplementary figures and images for: The sucrose non-fermenting 1-related kinase 2 gene SAPK9 improves drought tolerance and grain yield in rice by modulating cellular osmotic potential, stomatal closure and stress-responsive gene expression
Source: BMC Plant Biol. 2016 Jul 13;16:158. doi: 10.1186/s12870-016-0845-x (PMC4944446; doi:10.1186/s12870-016-0845-x)

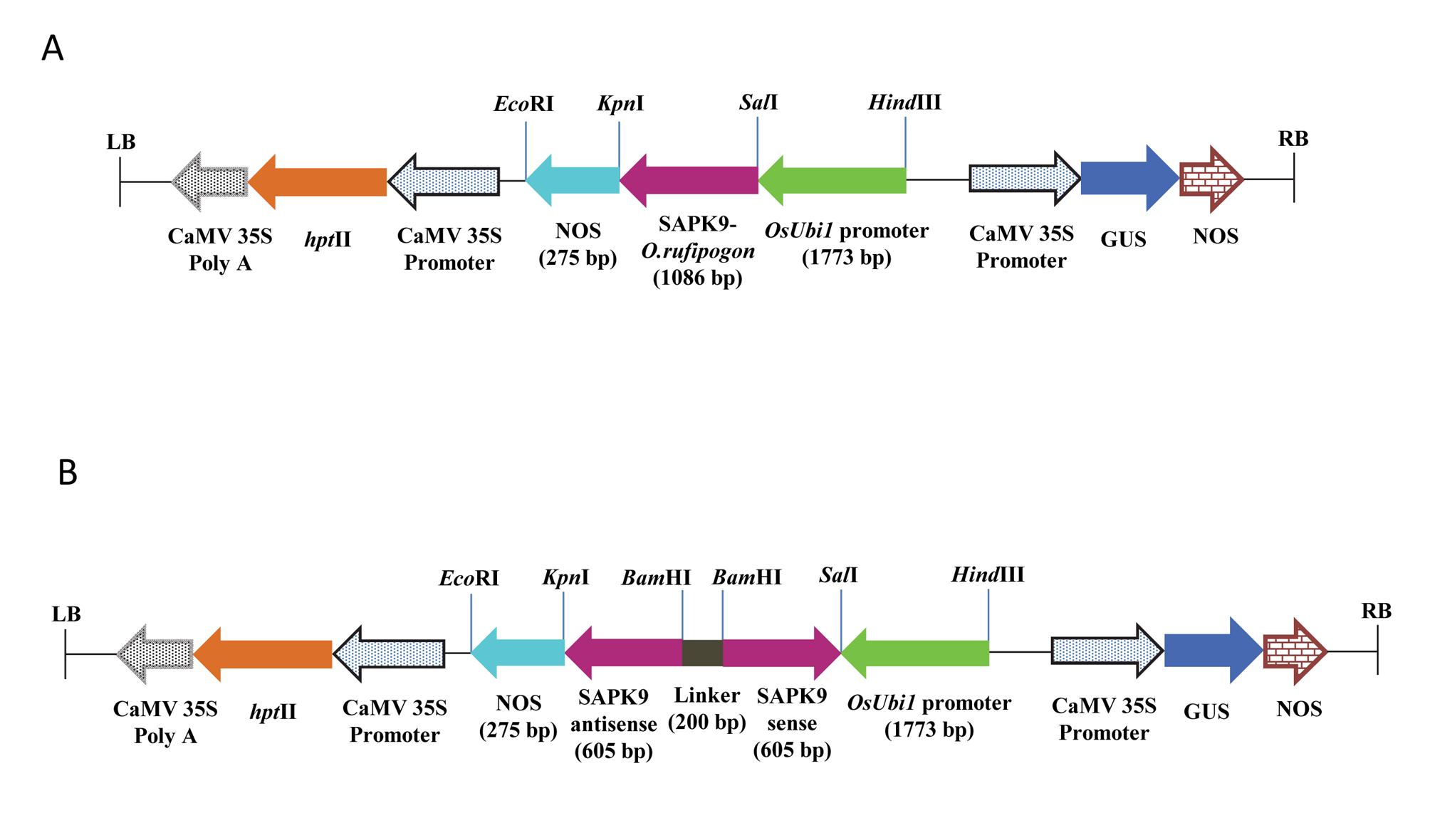

Supplement: Additional file 2: Figure S5. — Schematic representation of the genetic constructs based on pCAMBIA1301 plasmid used for Agrobacterium-mediated transformation of drought-sensitive indica rice cultivar IR20. (A) The gene overexpression (OE) construct of SAPK9 carrying 1086 bp CDS from O. rufipogon. The developed transgenic rice lines were designated as SAOE#1, 2, 3 etc. (B) The RNAi-mediated gene silencing (RNAi) construct of endogenous SAPK9 gene. The 605 bp 5′-part of SAPK9 CDS from O. rufipogon was cloned in sense and antisense orientation flanking an arbitrary 200 bp DNA linker. The developed transgenic rice lines were designated as RNAi#1, 2, 3 etc. (TIF 453 kb) [file 12870_2016_845_MOESM2_ESM.tif]

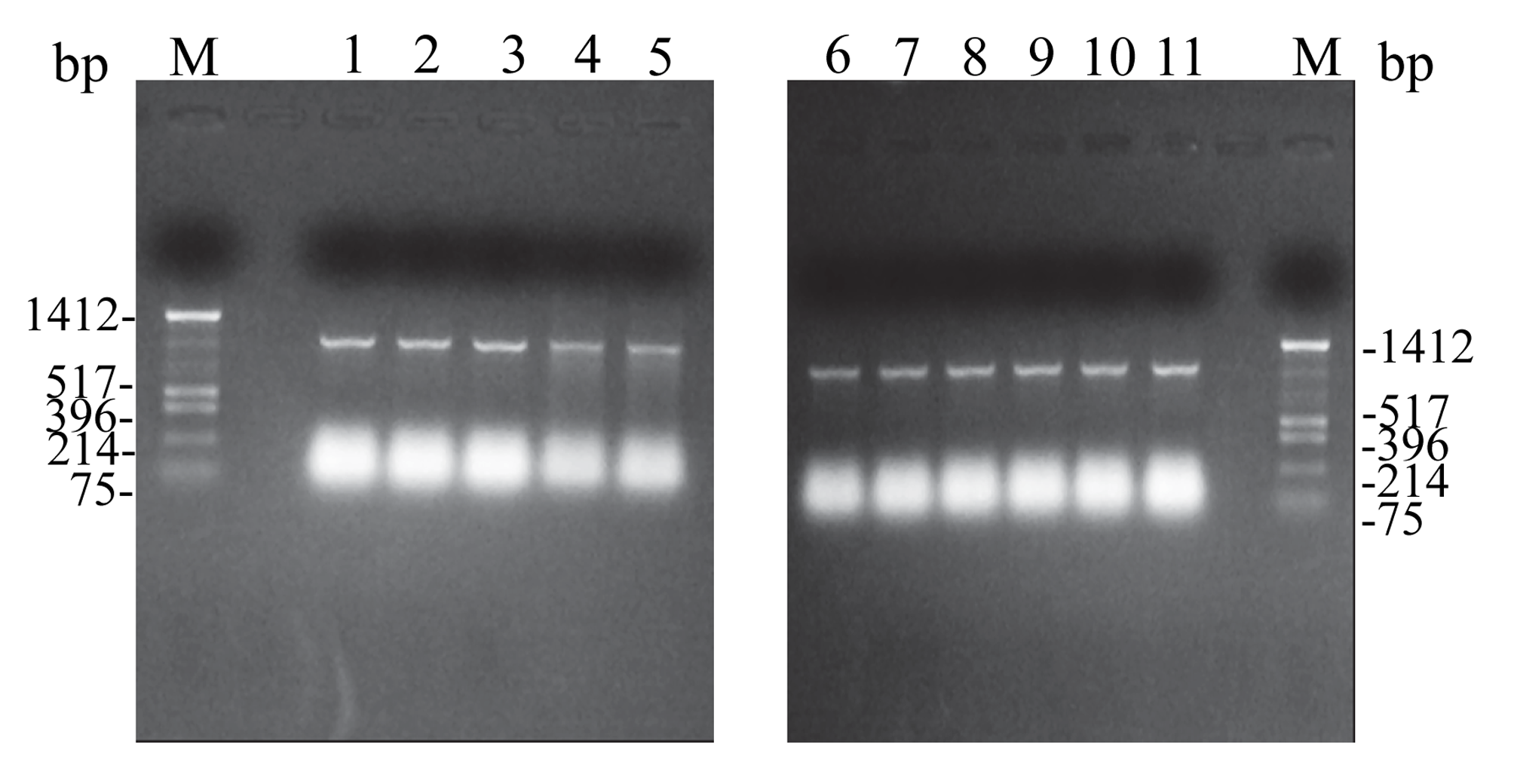

Supplement: Additional file 3: Figure S1. — PCR-amplified SAPK9 CDS from the chosen rice genotypes. Lanes 1-11 represents O. rufipogon, O. nivara, Nagina22, Manipuri, Vandana, Swarna, IR20, IR36, IR64, IR72, HRC300, respectively. Lane M- standard molecular weight marker. (TIF 1083 kb) [file 12870_2016_845_MOESM3_ESM.tif]

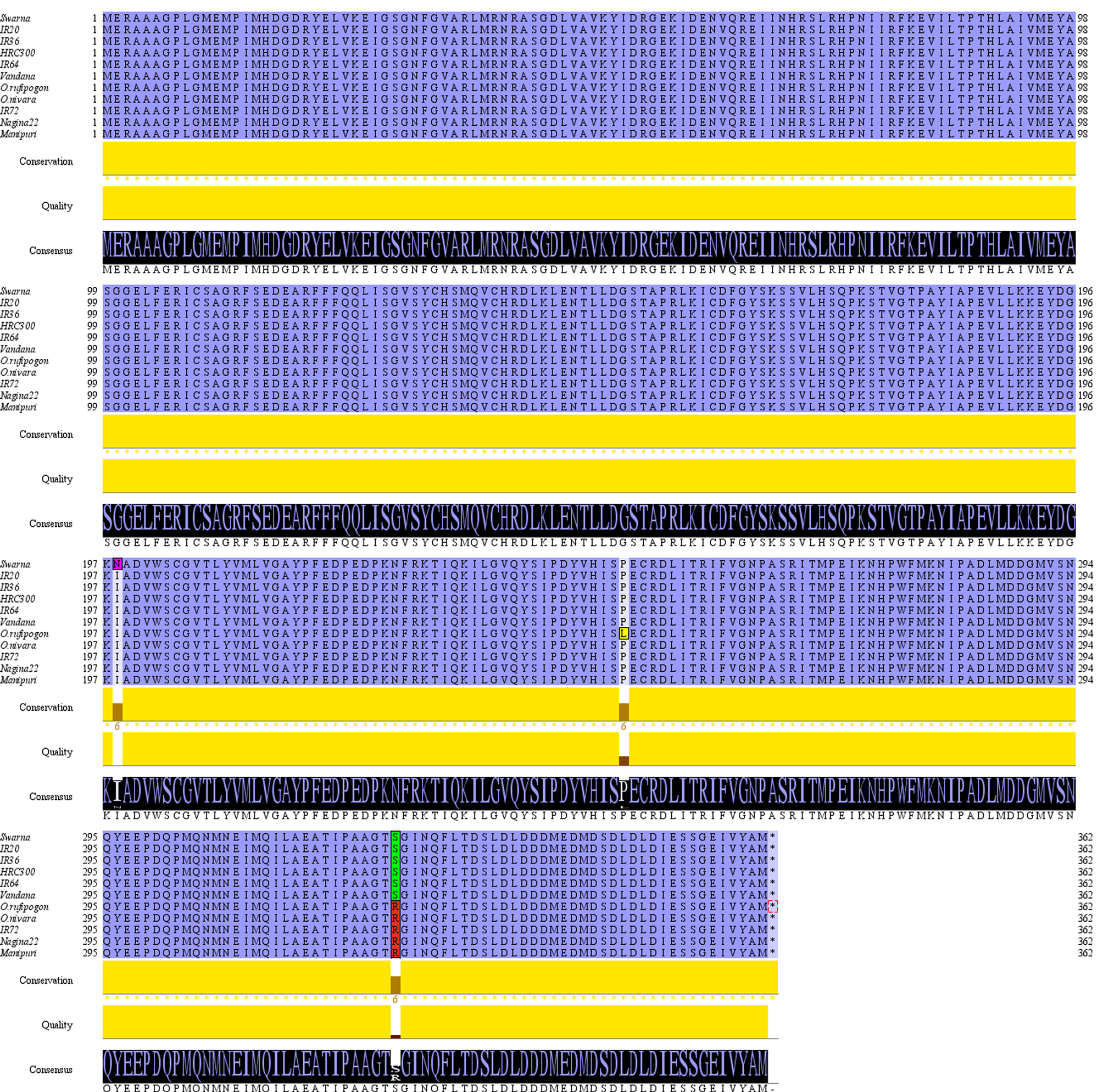

Supplement: Additional file 4: Figure S2. — Multiple sequence alignment for analysis of amino acid polymorphism in the SAPK9 CDS from the selected rice genotypes using Jalview software. (TIF 3333 kb) [file 12870_2016_845_MOESM4_ESM.tif]

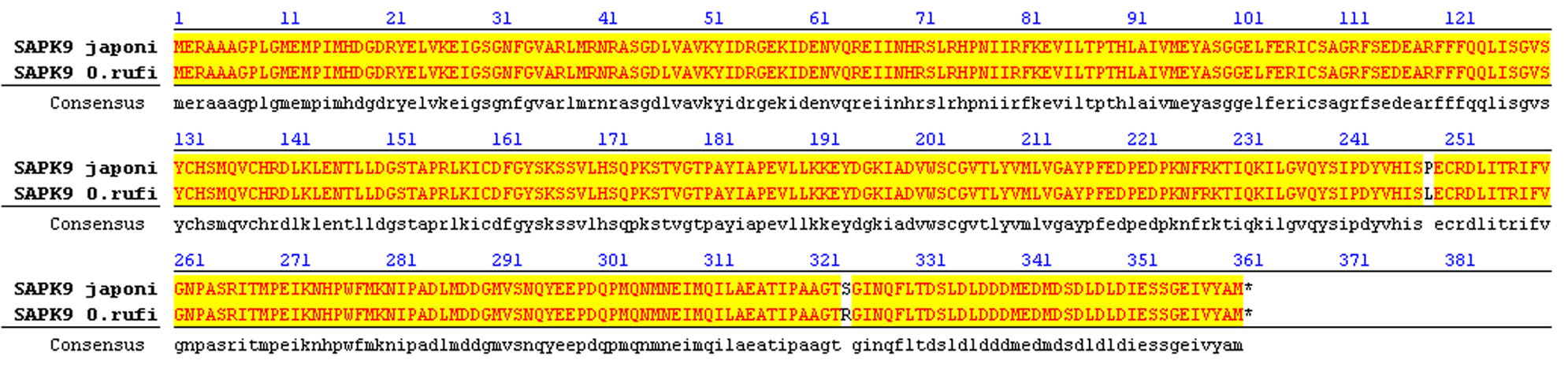

Supplement: Additional file 5: Figure S3. — Amino acid sequence alignment of the isolated SAPK9 CDS from wild rice Oryza rufipogon (accession no. KT387673) with the reported sequence of japonica rice cultivar (accession no.AB125310). (TIF 487 kb) [file 12870_2016_845_MOESM5_ESM.tif]

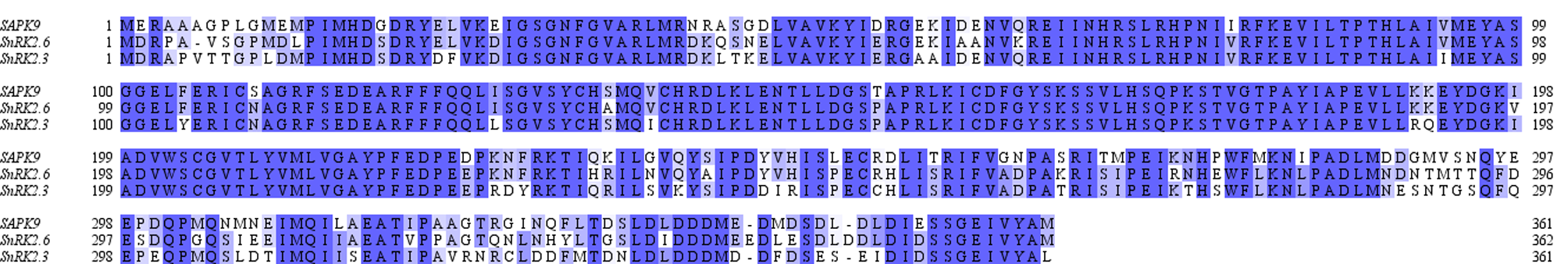

Supplement: Additional file 6: Figure S4. — Multiple alignments showing amino acid sequence similarity of SAPK9 with Arabidopsis OST1/SnRK2.6 (PDB ID: 3UC4) and SnRK2.3 (PDB ID: 3UC3). (TIF 644 kb) [file 12870_2016_845_MOESM6_ESM.tif]

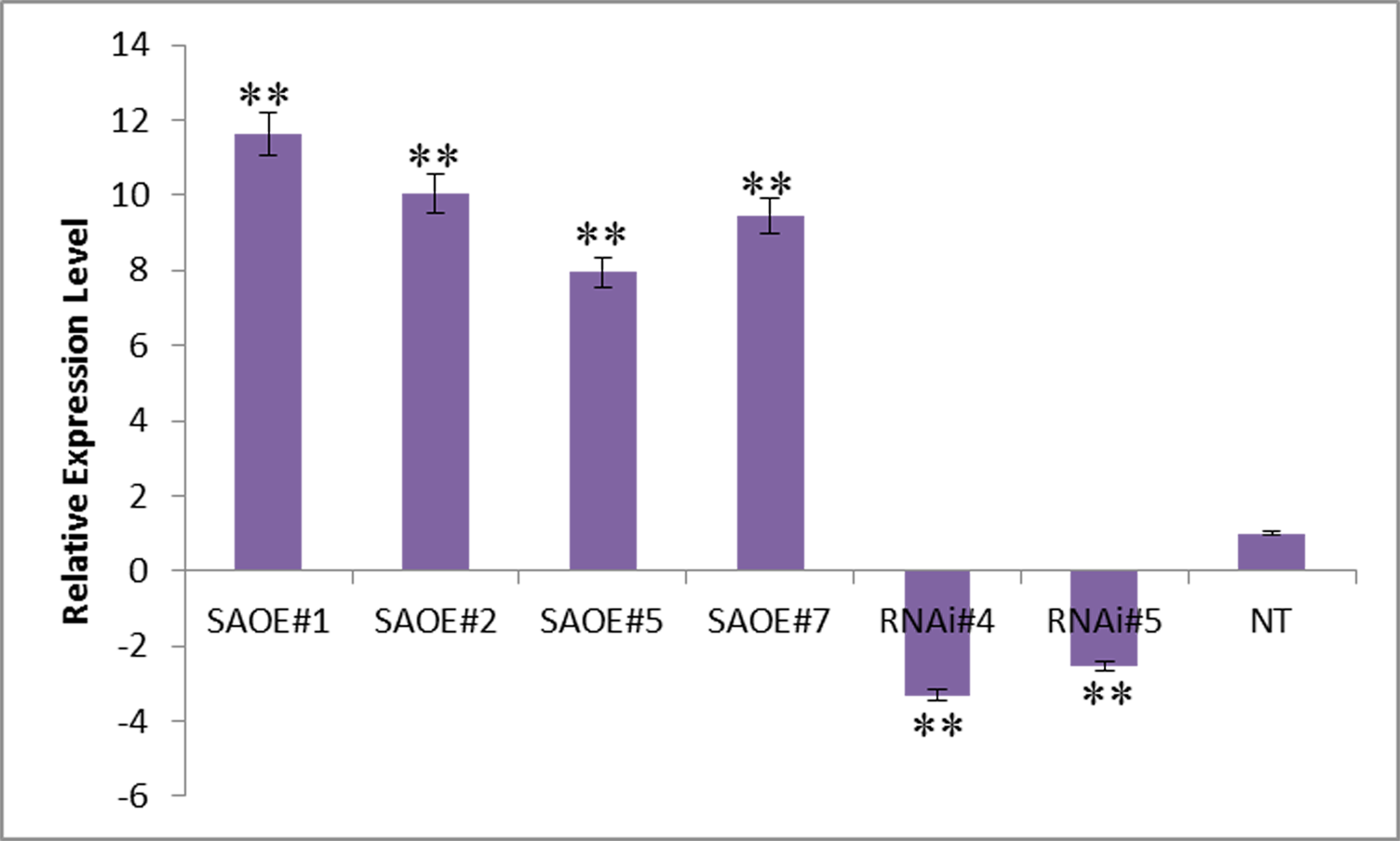

Supplement: Additional file 7: Figure S7. — The relative expression level of SAPK9 gene in reproductive stage (panicle initiation) was analysed through real-time PCR from leaf tissues of three sets of plants, i.e. OE, RNAi and NT plants. For internal reference, rice polyubiquitin1 (OsUbi1) gene was used. Error bars represent the mean ± SD of triplicate measurements. Student’s t-test was performed to find out statistically significant differences (**P < 0.01). (TIF 240 kb) [file 12870_2016_845_MOESM7_ESM.tif]

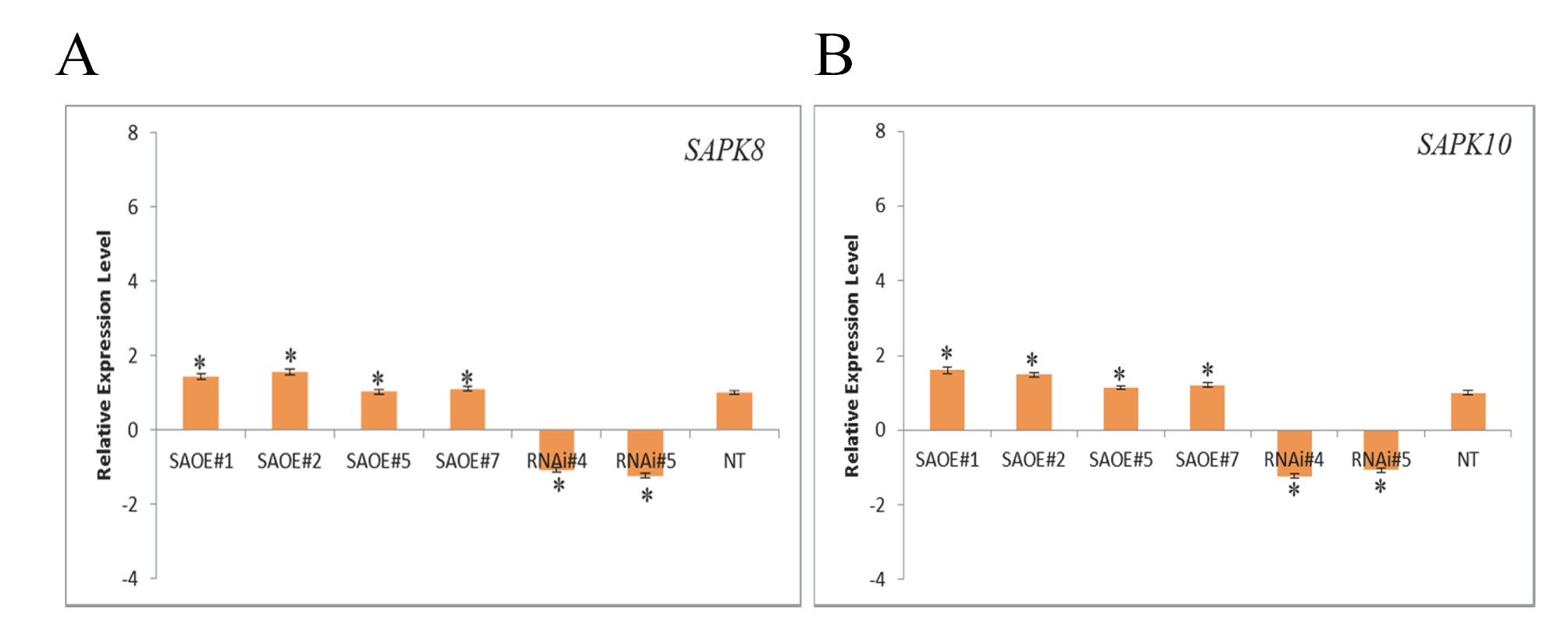

Supplement: Additional file 8: Figure S6. — Relative expression level of SAPK8 and SAPK10 gene in SAPK9 overexpressed (OE) and gene silenced (RNAi) transgenic rice lines. Analysis of real-time PCR depicting transcript level of (A) SAPK8 and (B) SAPK10 in leaf tissues of three sets of plants, i.e. OE, RNAi and non-transgenic (NT) plants under drought stress. For internal reference, rice polyubiquitin1 (OsUbi1) gene was used. Error bars represent the mean ± SD of triplicate measurements. Student’s t-test was performed to find out statistically significant differences (*P < 0.01). (TIF 339 kb) [file 12870_2016_845_MOESM8_ESM.tif]

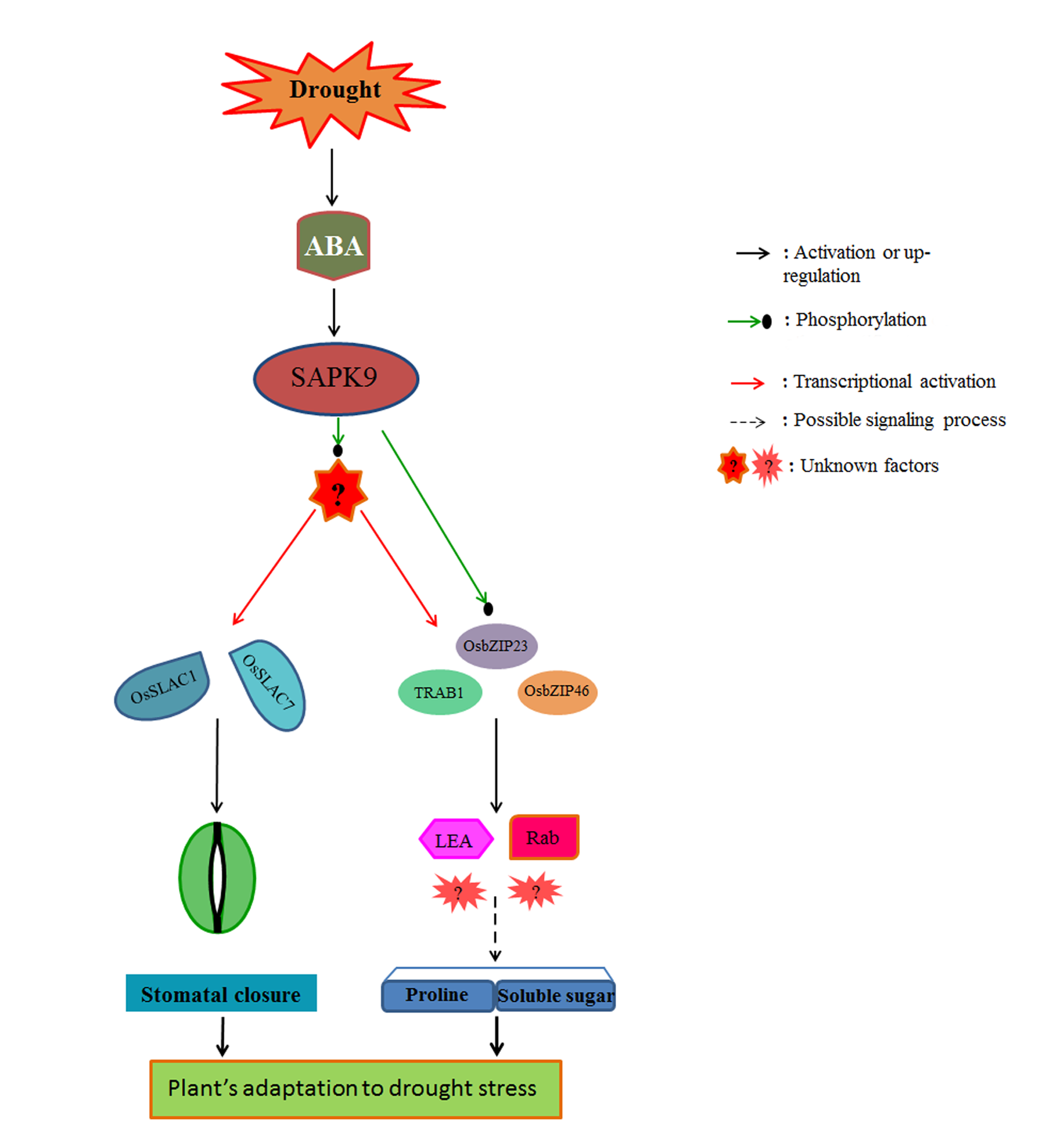

Supplement: Additional file 9: Figure S8. — Possible molecular mechanism of SAPK9-regulated drought stress tolerance in rice. Upon drought stress, the SAPK9 is activated by ABA signaling pathway, which in turn phosphorylates the bZIP transcription factors activating the expression of downstream genes involved in maintaining the physiological conditions for cellular homeostasis. Similarly, the SAPK9 also transcriptionally activates (might require an unknown factor) for upregulation of several hierarchically downstream genes including the anion channel genes leading to stomatal closure. (TIF 899 kb) [file 12870_2016_845_MOESM9_ESM.tif]
